# Prediction of the properties of sintered copper-graphite composites using response surface methodology

**DOI:** 10.1371/journal.pone.0357759

**Published:** 2026-09-11

**Authors:** Ankit, Vineet Kumar, Manvandra Kumar Singh, Gopal Ji, Swayam Prakash Kutar, Sandeep Kumar Gupta, Raj Bahadur Singh, Rohit Kumar Singh Gautam, Jitendra Kumar Katiyar

**Affiliations:** 1 Department of Mechanical Engineering, Kamla Nehru Institute of Technology, Sultanpur, Uttar Pradesh, India; 2 Department of Mechanical Engineering, Graphic Era (Deemed to be University), Dehradun, Uttarakhand, India; 3 Department of Metallurgical Engineering, Indian Institute of Technology (BHU) Varanasi, Varanasi, Uttar Pradesh, India; 4 Department of Material Science and Engineering, National Institute of Technology Hamirpur, Hamirpur, Himachal Pradesh, India; 5 Department of Mechanical Engineering, Teerthanker Mahaveer University, Moradabad, Uttar Pradesh, India; 6 Manipal Institute of Technology, Manipal Academy of Higher Education, Manipal, India; UNICAMP, University of Campinas, BRAZIL

## Abstract

The present study aims to predict the density and hardness of copper-graphite composites prepared by powder metallurgy using response surface methodology. Copper-graphite composites were investigated for phase composition and microstructure properties. Density and hardness values were assessed using a central composite design, followed by RSM-based optimization. Sintering temperature, holding time, and graphite weight percentage were used as process parameters, while density and hardness were the response variables. The significance of process conditions was assessed using ANOVA. A quadratic regression model was fitted to estimate the response variable values. It was found that the density and hardness values were highest in the composite containing 3.06 wt.% graphite, sintered at 944°C for 1.5 h. The calculated values demonstrated excellent agreement with the experimental data, exhibiting negligible errors.

## 1. Introduction

Composite materials have seen significant attention in recent years owing to their enhanced properties and broad range of industrial applications. Amongst them, metal matrix composites (MMCs) offer a unique blend of electrical performance, mechanical strength, wear resistance, and corrosion resistance. MMCs comprise ceramic reinforcements distributed within a metal or alloy matrix [1–4]. The behavior, such as mechanical, physical, tribological, etc., properties of MMCs are significantly impacted by the choice of matrix, type and amount of reinforcement used, as well as the processing techniques employed. Copper (Cu) and Cu- based alloys are frequently used as matrices in MMCs owing to their exceptional ductility, thermal and electrical conductivity, and ease of processing. These characteristics make copper an ideal contender for electrical and thermal management applications such as bearings, bushings, and electrical brushes.

Graphite, recognized as an effective solid lubricant, has been widely studied as a reinforcement in copper-based matrices because of its layered structure, which allows easy shearing between layers under little force, thereby improving the self-lubricating behavior of the composite [5]. Therefore, copper–graphite composites are increasingly employed in automotive and electrical uses such as carbon brushes, pantographs, and other sliding components, where both conductivity and wear resistance are critical [6,7]. Researchers have focused on developing graphite-reinforced copper composites and investigating their various properties. Liu et al. [8] fabricated graphite–Cu composites using graphite flakes of varying particle sizes and observed an increase in thermal conductivity; however, the soft nature of graphite (Gr) led to a decrease in composite strength. Jhulan Kumar et al [9] synthesized copper composites reinforced with graphite and found that incorporating 5 wt.% graphite led to the largest increase in compressive strength, along with a notable improvement in wear performance. Varol et al [10] fabricated copper-graphite nanocomposites by means of the powder metallurgy technique and revealed that as the graphite concentration increased, both hardness and wear resistance of the composites decreased.

The powder Metallurgy (PM) technique has proven to be a potent method for producing copper-based composites compared with other techniques. It enables precise control over composition, microstructure, and reinforcement distribution, as well as the production of complex-shaped products with minimal waste [11–17]. According to Suryanarayana, mechanical alloying through PM facilitates a uniform dispersion of reinforcement particles due to repeated deformation, cold welding, and fracture during milling [18]. Zuo et al. [19] fabricated copper/graphite composites using various sintering methods and found that microwave pressure sintering improved the composites’ overall properties. Ankit et al. [20] developed Cu-graphite-TiC composites and found that utilizing graphite reduced the composite’s hardness, whereas titanium addition improved both hardness and wear performance. When preparing a composite via powder metallurgy, it is necessary to determine the sintering parameters that achieve the desired composite properties. Several statistical models developed by researchers may achieve this optimization. Numerous modeling techniques, namely, Response Surface Methodology (RSM), Machine Learning, Taguchi methods and Artificial Neural Networks (ANN), are progressively being employed to augment parameters like sintering time, sintering temperature or the parameters affecting the mechanical, frictional and wear behavior of composites, through statistical analysis performed in Python, Minitab, Design of Experiments (DOE), or MATLAB [21,22]. Design of Experiments (DOE) is a structured statistical framework that facilitates the design, execution, and analysis of experimental studies, aiding the evaluation and understanding of how input constraints affect a given response. RSM is an assembly of statistical and mathematical procedures to assess the influence of input variables on output responses, aimed at identifying optimal conditions for material performance [23]. Response Surface Methodology works by developing a polynomial equation that precisely represents the relationship between the input factors and the output, using statistical data from experimental results. Recent scientific studies have widely utilized RSM to assess the significance and impact of input factors on the resulting output responses. The input factors that lead to enhanced material properties should be identified, which would, in turn, help with material and resource reduction. Various studies have been carried out to optimise different properties (mechanical and tribological) of composites with the help of RSM. Kumar and Devaraju [24] utilized RSM to optimise multiple properties—such as hardness, density, specific wear rate, etc. of CRS/SiC-reinforced aluminium composites. Magibalan et al [25] effectively applied RSM to enhance the wear performance of aluminium composites reinforced with fly ash. Vineet et al [26] effectively applied both RSM and ANN methods to augment the wear behavior of ZA/ZrB_2_ composites.

Based on the literature review, RSM can be efficiently applied to predict properties with respect to various sintering parameters of a composite fabricated by powder metallurgy, thereby enhancing performance and maximizing output benefits. However, limited research has been reported specifically on copper-graphite composites, with RSM modeling to enhance mechanical properties. Hence, the current study focuses on developing a statistical model of copper-graphite composites by varying graphite content. Sintered density and hardness of the composites were analyzed through central composite design (CCD) coupled with response surface methodology (RSM), considering sintering temperature, holding time, and reinforcement (graphite) content as the key input factors. Analysis of Variance (ANOVA) was also conducted to determine the interactive effects of input parameters on the density and hardness of the fabricated composites. An integrated approach was adopted by correlating experimental results with statistical Response Surface Methodology (RSM) to develop predictive relationships for the densification and hardness responses as functions of the process parameters (sintering temperature and holding time) in a Cu–Gr composite. In contrast to earlier research, this study focuses on prediction and validation within the processing range.

## 2. Materials and methodology

### 2.1 Fabrication of composites

Powder metallurgy (PM) is a cost-effective and efficient fabrication technique used to produce metal and composite components with precise dimensions. In this study, PM is utilized to fabricate the graphite-reinforced copper composites. Commercially pure copper powder (purity ≥ 99.50%) with an average particle size of 45 µm was used as the matrix material. The graphite powders were used as a reinforcement material having a purity greater than 99%, with particle sizes ranging from 7–11 µm. The copper and graphite powders were weighed using a balance to achieve the desired weight percentages. The parameters for the preparation of composites via powder metallurgy are given in Table 1.

**Table 1:** Powder metallurgy steps employed.

Steps	Details
Milling Method	Ball milling (RETSCH PM400)
Milling Time	5 ± 2 min hours (dry, intermittent)
Media & Speed	WC balls (10 mm) & 300 ± 5 rpm
Ball-to-Powder Ratio	5:1
Compaction	Die-punch, Pressure- 700 ± 10 MPa
Furnace Used	Nabertherm RHTH 120–300/18
Sintering temperature	900 ± 5°C −1000 ± 5°C, heating rate: 5 °C/min in Argon
Sintering time	60 ± 3 min −120 ± 3 min
Final Shape	Pin (6 mm diameter × 8 mm length)

**Fig 1:**
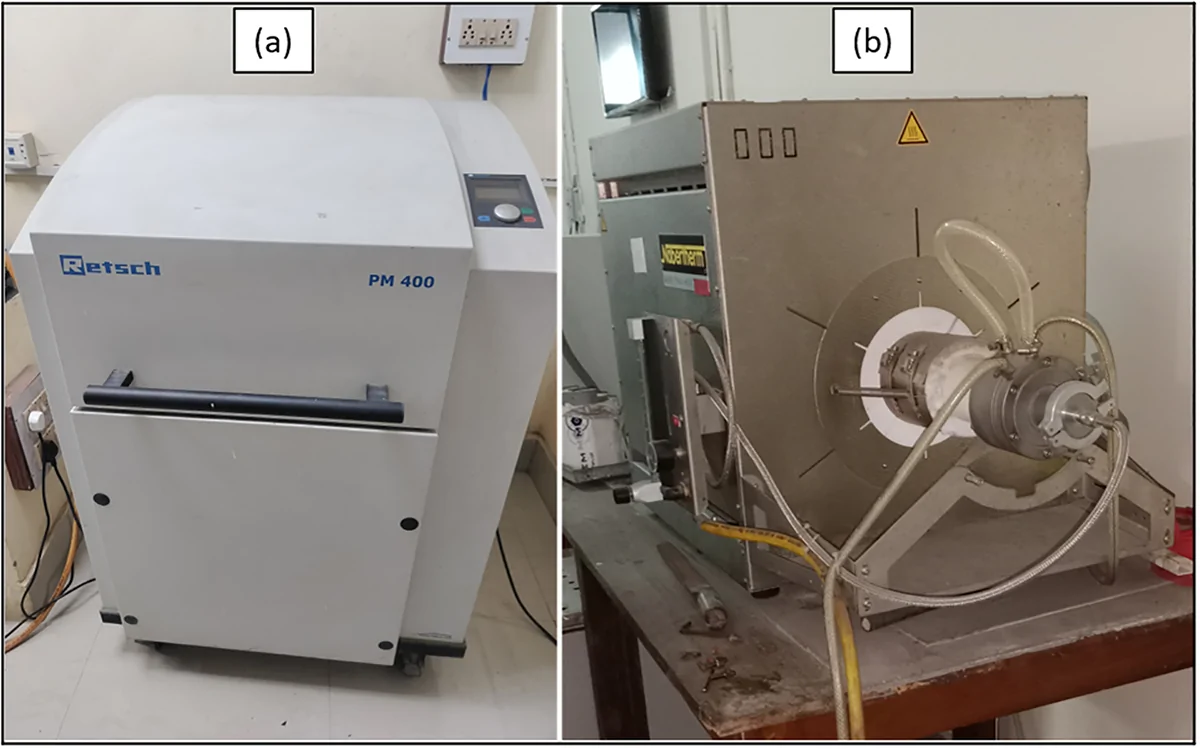
(a) RETSCH PM400 milling machine and (b) Nabertherm tube furnace.

After compaction in the die-punch setup, the green densities of compacts were identified using Archimedes’ principle. Furthermore, the composites were fabricated by sintering in an argon atmosphere at 900 °C, 950 °C, and 1000 °C. The holding times were 1, 1.5 and 2 h, respectively. The ball milling machine employed was a RETSCH PM400 (Fig 1(a), and the tube furnace used to sinter the composites is shown in Fig 1(b).

Three copper-graphite composite compositions were prepared with graphite contents ranging from 2.5 to 7.5 wt.%. The nomenclature of the composites was G1 (copper with 2.5 wt.% graphite), G2 (copper with 5 wt.% graphite) and G3 (copper with 7.5 wt.% graphite), respectively.

### 2.2 Characterization and testing of composites

Microstructural characteristics of the fabricated samples were examined using optical microscopy and scanning electron microscopy (SEM). For microstructural analysis, samples were prepared by progressive grinding with silicon carbide (SiC) abrasive papers of grit sizes 400–2000, followed by polishing on a cloth using alumina (Al_2_O_3_) powder. The samples were subsequently examined using a DM 1750M Leica microscope and an EVO-SEM MA15/18 scanning electron microscope (Carl Zeiss). Phase identification of the composites was carried out by X-ray diffraction (XRD) using a Rigaku MiniFlex diffractometer with Cu–Kα radiation (λ = 1.5406 Å).

Their density greatly influences the hardness of composites synthesized via powder metallurgy (PM). Archimedes’ principle was employed to measure the sintered densities of the prepared samples in accordance with ASTM B962. The sintered density of the samples was measured five times for each composite. The density range for each composite is shown as error bars in the respective (Figs 5–7). The hardness measurement of composites was also carried out in line with the ASTM E92 standard. An LM248AT Leco microhardness tester was employed to determine the Vickers hardness of composites. Hardness testing was conducted on polished samples under a 0.5 kg load with a 30-second dwell time. To ensure reproducibility of the results, the test was performed 7 times at various locations on each polished sample, and the mean was used to determine the average hardness.

### 2.3 RSM-based modelling of density and hardness

Design of Experiments (DOE) is a powerful statistical technique used to systematically plan, conduct, and analyze experiments to evaluate the effects of multiple factors on response variables efficiently. The sintered density and hardness of the composites were predicted using Design-Expert software (Version 13.0). Each experimental condition defined by the Central Composite Design (CCD) corresponded to one fabricated specimen, on which repeated density and hardness measurements were performed to evaluate the measured properties. Based on the statistical analysis performed with the software, a quadratic model was found to be the most suitable for describing the relationship between process variables and responses. RSM is a vital mathematical technique for designing experiments, developing models, and optimizing responses to achieve the best possible outcomes. Central Composite Design (CCD) is a commonly used experimental design technique within Response Surface Methodology (RSM) that helps model and optimise complex interactions between input variables and the desired output. CCD includes three stages: design of the experiment, model development (fitting the experimental data to a suitable mathematical model) and interpretation of the outcomes. Central Composite Design (CCD) is commonly used to develop second-order (quadratic) polynomial models in Response Surface Methodology (RSM). The given work utilizes CCD to predict the response outputs, such as density (sintered density) and hardness, as a function of input variables. The input factors selected for the study—wt.% of Graphite (A), Sintering temperature (B), Holding time(C) are also referred to as independent factors. Table 2 presents the factors, along with their respective upper and lower limits, used in planning the experiment.

**Table 2:** Experimental factors and levels used in RSM for composites.

Factor	Unit	-α	+α	Low (−1)	High (+1)
A: Graphite content	Weight %	2.5	7.5	2.5	7.5
B: Sintering temperature	°C	900	1000	900	1000
C: Sintering time	h	1	2	1	2

It is assumed that the response values fall within the specified range of these factor levels. In central composite design, the axial points are placed symmetrically around a central point, and the distance from the center to each axial point is called alpha (α). In face-centered CCD designs, the alpha (α) values range from +1 to −1. An alpha value greater than one (>1) signifies that the point is outside the cube, while α = 1 means the point exists on the surface of the cube; and if α < 1, the point is situated within the cube [24]. Also, the sequence in which the experimental runs are executed is randomized.

## 3. Results and discussion

### 3.1 Phase examination

**Fig 2:**
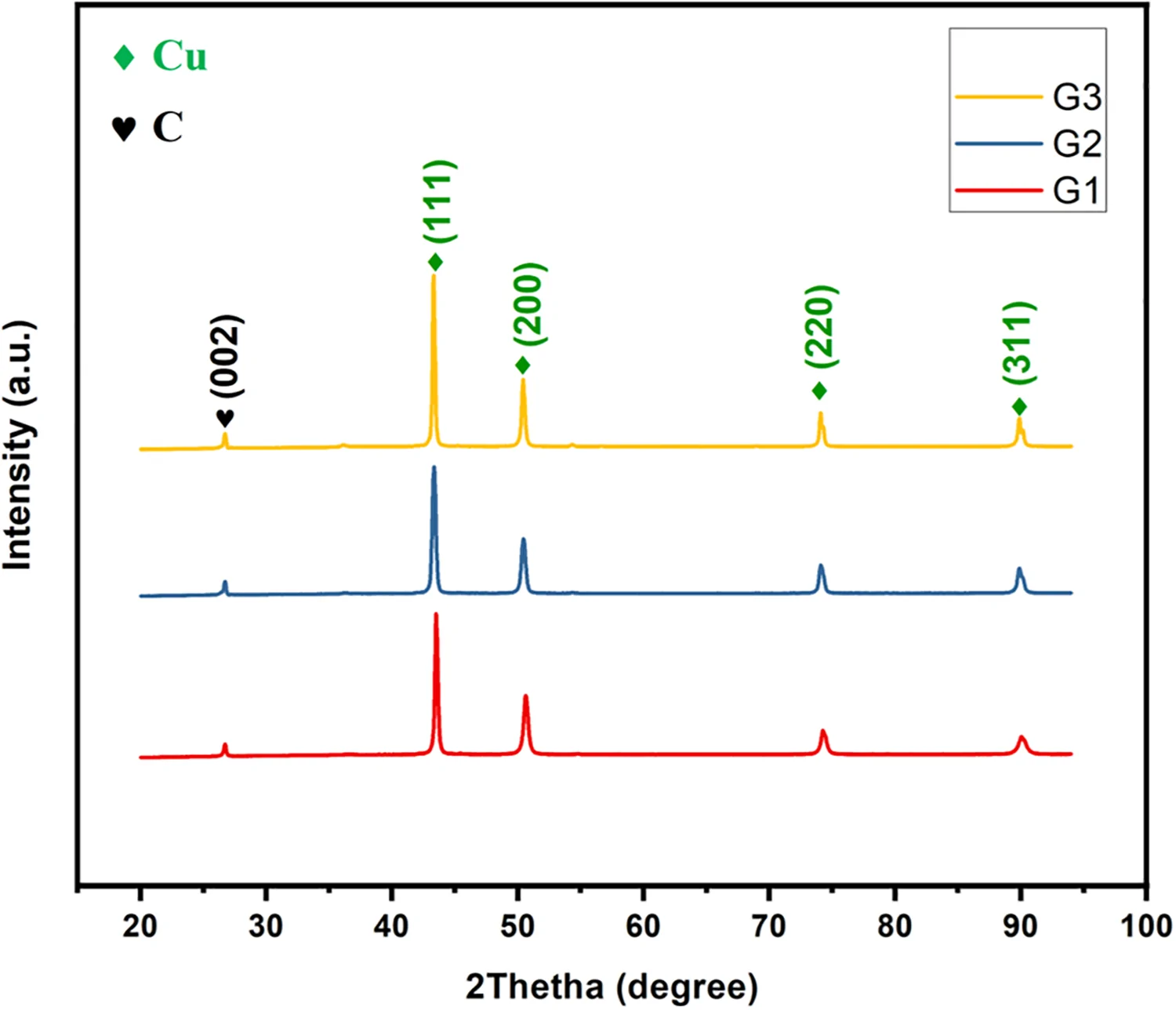
XRD patterns of the various composites.

The bulk sintered composites were tested for phase identification using X-ray diffraction. Five samples of each composite were tested to ensure reproducibility and consistency of the results. The XRD pattern of the sintered composites with varying graphite content (G1, G2 and G3) at a 950°C sintering temperature and 1.5 h sintering (holding) time is shown in Fig 2. The composites were successfully fabricated, and the formation of the desired phases was verified. Composites displayed distinct peaks for copper (Cu) and carbon (C), indicating the presence of graphite within the material. No additional phases were observed in the pattern, suggesting that no reaction occurred among the matrix copper, graphite reinforcement, and ambient oxygen. Each peak was assigned to its respective crystallographic plane and illustrated in the pattern. The peaks of pure copper exactly matched with JCPDS File No. 4–836 with corresponding reflection planes (111), (200), (220), and (311), respectively [27]. The diffraction peak of graphite corresponding to the (002) plane is in accordance with JCPDS No. 41–1487 [28].

### 3.2 Microstructural analysis

**Fig 3:**
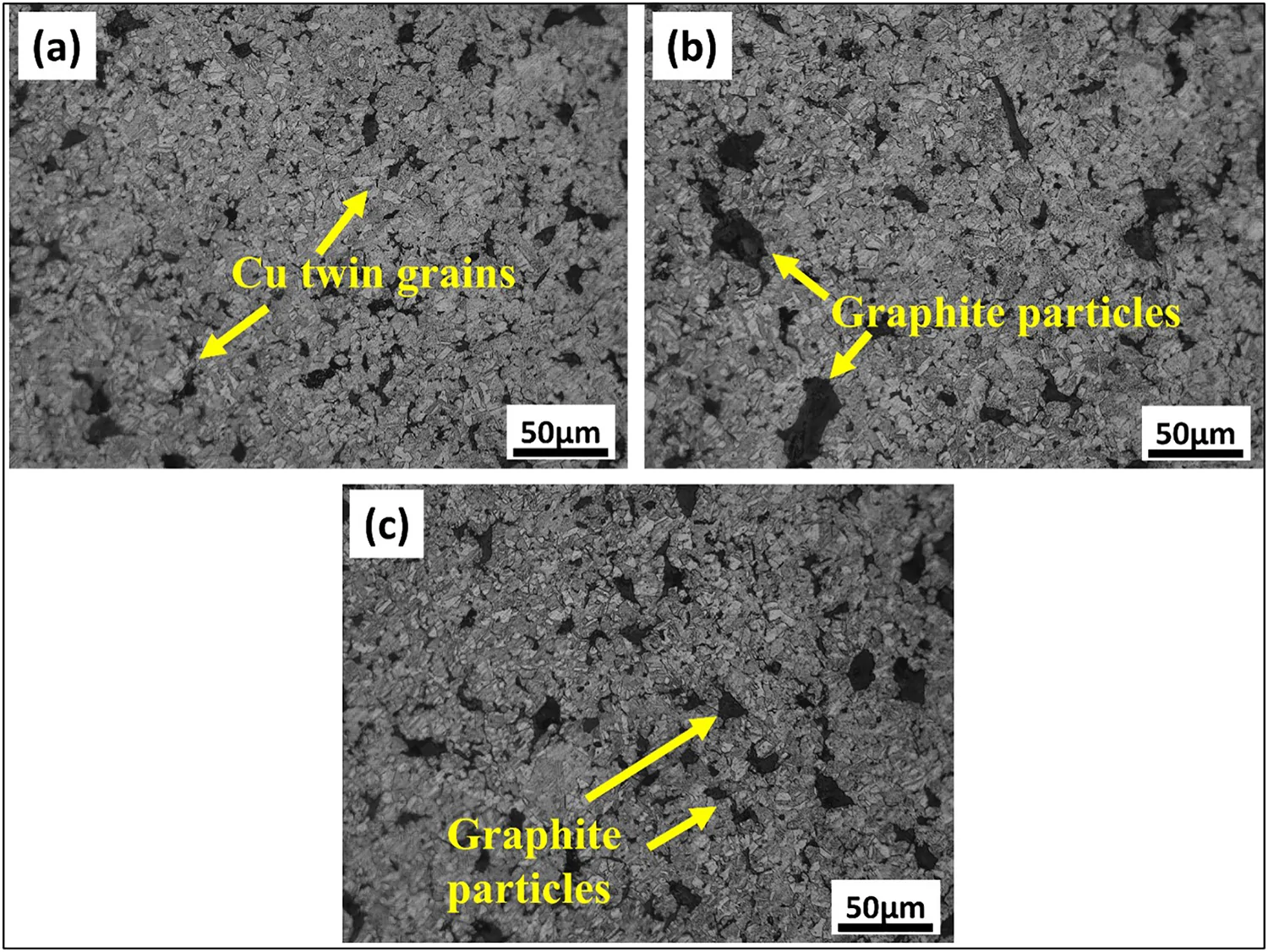
Optical images of (a) G1, (b) G2, and (c) G3 composite.

**Fig 4:**
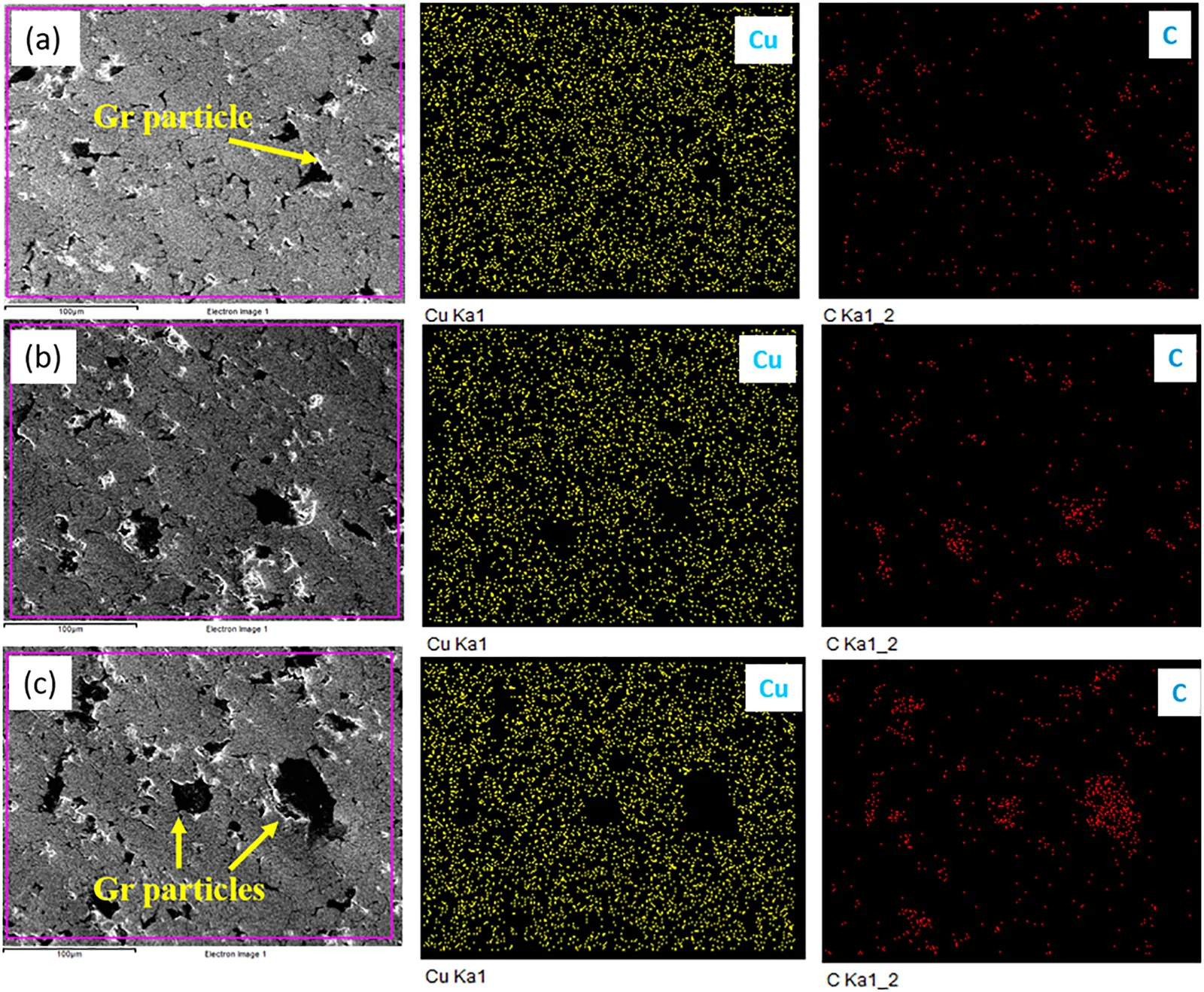
SEM of (a) G1, (b) G2, and (c) G3 composite having EDS elemental mapping.

The optical images of composites after etching are depicted in Fig 3. The etchant solution was made using 10g Fe_3_Cl, 10 ml HCL, and 100 ml distilled water. Three samples for each composite were examined to assess the reproducibility of the optical observations, and consistent microstructural features were observed across all samples. The optical images of all composites (G1-G3) show well-defined twin grains in the copper matrix, with a mostly uniform distribution of graphite particles. However, some agglomeration may be due to the finer particle size. The SEM micrographs of a composite show the interfacial bonding between the reinforcement particles and the matrix phase. It enables understanding of the dispersion of added Gr particles within the matrix, thereby improving composite properties [29,30]. SEM images from three independently prepared samples for each composite were analyzed. Fig 4 shows SEM of the composites together with the corresponding EDS elemental maps. Elemental mapping was performed to detect the presence of copper (Cu) and graphite (C) in the composites. The mapping was done over a defined square region in each SEM image. EDS elemental mapping suggests a relatively uniform distribution of graphite within the analyzed region of the copper matrix, with no obvious large-scale agglomeration observed. Also, an increase in the number of carbon (C) elemental dots was observed, indicating a higher graphite particle content in the composites. More elemental dots of carbon (C) were seen in the G3 composite than in the G1 composite. SEM analysis further reveals enlarged, interconnected pores that may be associated with the inherently limited wettability of the Cu–graphite system.

### 3.3 Interaction effects of sintering parameters and graphite content on density and hardness

**Fig 5:**
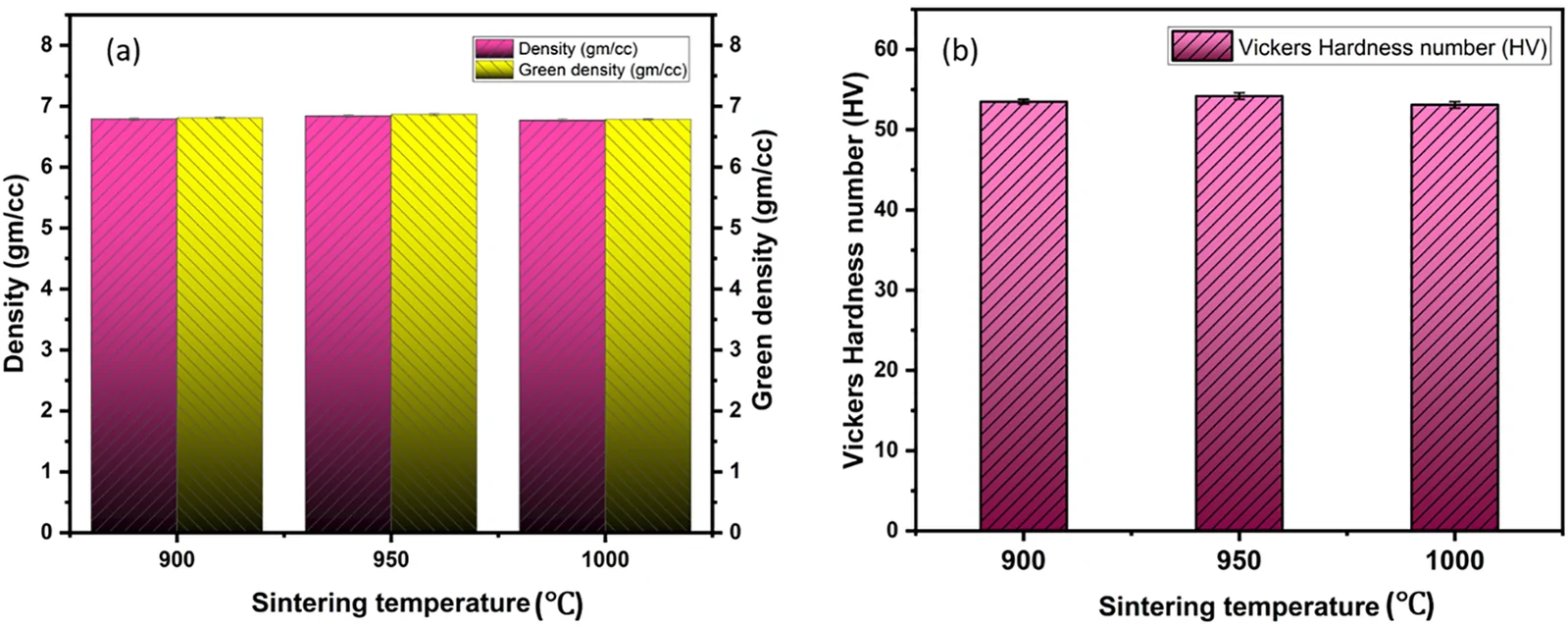
Influence of sintering temperature of G2 composite at constant holding time of 1.5 hr on (a) density, green density and (b) hardness.

**Fig 6:**
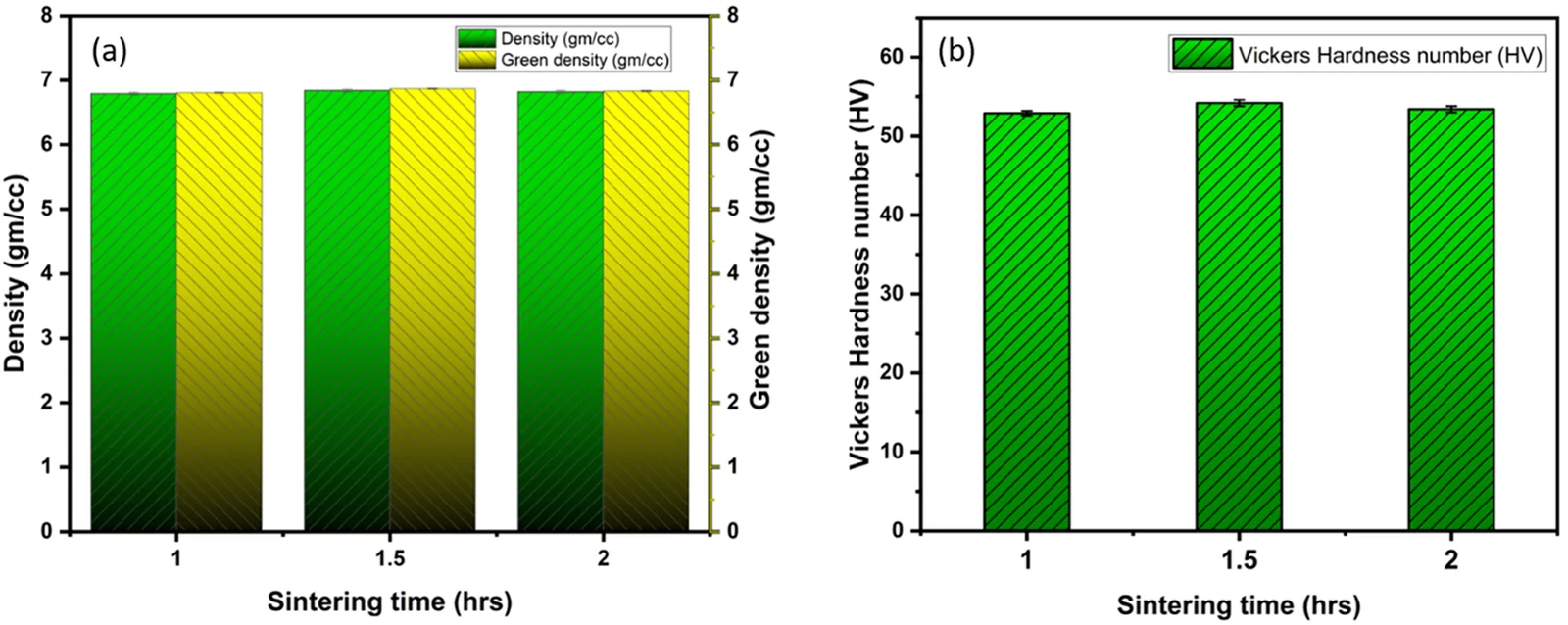
Influence of sintering time of G2 composite at constant sintering temperature of 950°C on (a) density, green density and (b) hardness.

**Fig 7:**
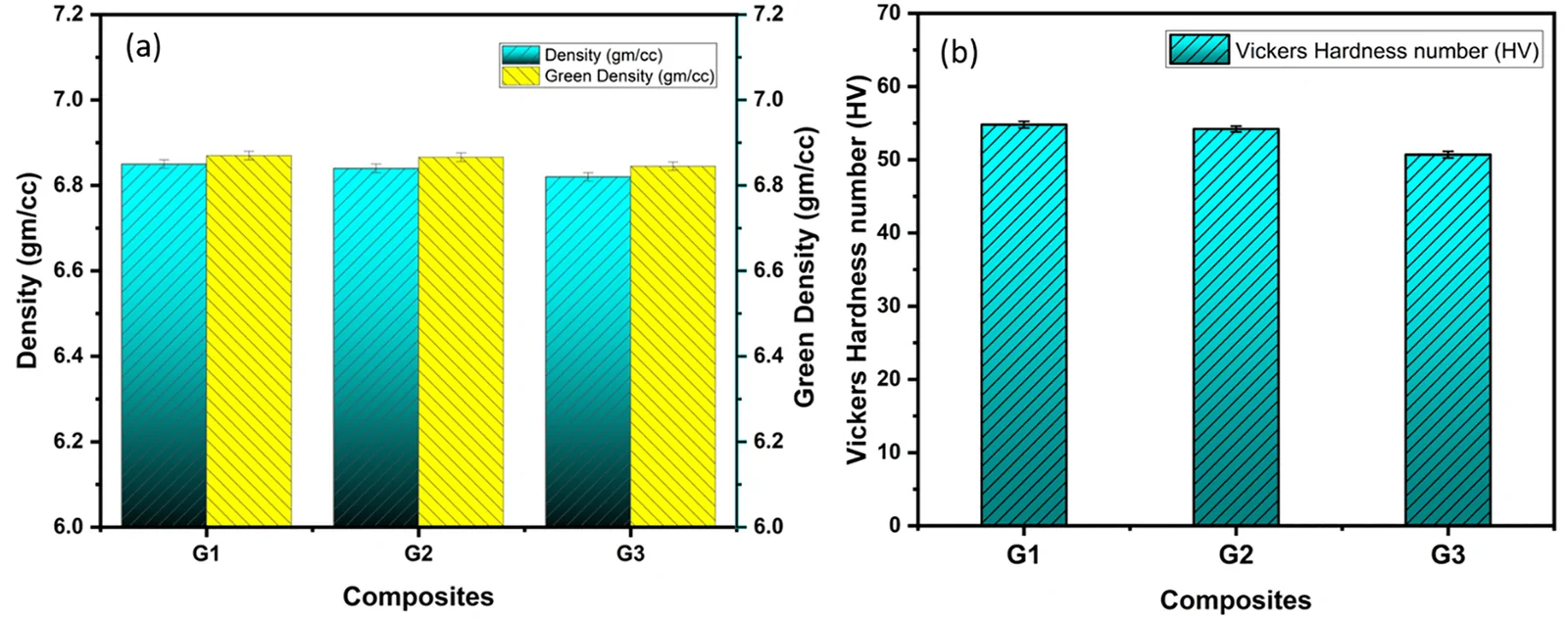
Influence of graphite percent at sintering temperature of 950°C and 1.5 hr sintering time on (a) density, green density and (b) hardness.

It is crucial to understand how sintering temperature and holding time affect the properties of composites produced by the PM route (density and hardness). Influence of sintering temperature, sintering (holding) time and reinforcement (graphite) content on the green density, density and hardness of the fabricated composites is presented in Figs 5–7. The density of all composites after sintering was lower than their green density across all parameters, possibly due to inadequate bonding between the reinforcements and the copper matrix, which may lead to voids. Fig 5 illustrates the variation in green density, density, and hardness of the G2 composite with sintering temperature at a fixed holding time of 1.5 hours. It is seen that at a sintering temperature of 950°C, maximum sintered density occurs owing to the enhanced diffusion and particle bonding between atoms of powder particles. Also, elevated temperatures increase the surface energy of the particles, enhancing atomic mobility and promoting neck formation between neighbouring powder particles. Consequently, a dense and well-bonded structure is obtained. However, a further rise in temperature significantly decreases the density, primarily due to grain enlargement in the copper matrix. It is also observed that as the temperature rises, the VHN values increase due to improved bonding among the powder particles. However, a further increase in sintering temperature causes coarsening of grains, which results in a decline in the composite’s hardness.

Fig 6 suggests that a longer holding time enhances the density and hardness of the composite up to 950°C, which may indicate proper bonding among the powder particles during the higher holding period. However, when the holding time was raised to 2 h, both density and hardness decreased, which can be attributed to grain growth in the copper matrix and an increase in pore size during prolonged sintering. The density and hardness show slight percentage decreases of 0.3% and 1.5%, respectively, as the holding time is increased from 1.5 h to 2 h.

Fig 7 depicts changes in density and hardness as graphite content varies. It illustrates the density and hardness values of composites G1, G2 and G3 sintered at 950°C with a dwell time of 1.5 h. It was found that the sintered density of the copper graphite composite significantly decreased with the graphite content, which can be attributed to the considerable difference in the densities of graphite (2.25 g/cm^3^) and copper (8.96 g/cm^3^) matrix. As the amount of graphite increases from G1 to G3 composite, the density is reduced, with G3 composite having the minimum density of 6.82 g/cm^3^. It should be noted that the absolute density of Cu, the density difference between copper and graphite, intrinsically influences graphite composites. Consequently, a reduction in measured density with increasing graphite content is expected from the rule of mixtures and should not be interpreted solely as a decrease in densification efficiency. Therefore, the density response should be viewed as the combined effect of composition and processing parameters. From a physical standpoint, the consolidation behavior is more appropriately represented by relative density, which accounts for the theoretical density for each composition. Thus, the optimization of the present system should be interpreted as achieving improved densification and mechanical performance within the investigated compositional range, rather than maximizing the absolute density. In addition to the intrinsic density difference between copper and graphite, factors such as graphite agglomeration, reduced wettability, and pore formation during sintering may further influence the achieved densification [31–34]. Also, the hardness of the composite decreases as the Gr % increases. The composite exhibits decreased hardness owing to the addition of graphite, which is inherently softer than copper. The hardness of G3 composite is reduced by 7.07% and 6.4% in comparison with the hardness of G1 and G2 composites, respectively.

### 3.4 Statistical analysis and prediction of density and hardness using response surface technique

#### 3.4.1 Central Composite Design (CCD).

The experimental design matrix was constructed using the Central Composite Design (CCD) technique of RSM, considering three levels for each input parameter. A total of 20 experimental iterations were carried out as per the CCD matrix, and the responses measured were ‘density’ and ‘hardness’ of the composite. The CCD included multiple replicated center-point experiments (5 wt.% graphite, 950°C, and 1.5 h) to estimate the experimental (pure) error and to assess the adequacy of the developed regression models through the lack-of-fit test. Table 3 presents the design matrix obtained from the CCD, incorporating the input factors (composition, temperature, and holding time) and the corresponding responses (density and hardness). The adequacy and reliability of the developed model were verified through analysis of variance (ANOVA) for both density and hardness responses [35].

**Table 3:** DoE varying with input parameters and output (response).

Std	Run	Input parameter 1	Input parameter 2	Input parameter 3	Output 1	Output 2
		A: Composition	B: Temperature	C: Holding/ Sintering time	Density	Hardness
		wt.%	°C	h	g/cc	VHN
7	1	2.5	1000	2	6.765	52.4
8	2	7.5	1000	2	6.74	48.4
17	3	5	950	1.5	6.84	54.2
16	4	5	950	1.5	6.84	54.2
13	5	5	950	1	6.8	53.22
4	6	7.5	1000	1	6.72	48.7
1	7	2.5	900	1	6.77	53.4
3	8	2.5	1000	1	6.75	52.8
14	9	5	950	2	6.82	53.4
2	10	7.5	900	1	6.735	48.9
6	11	7.5	900	2	6.755	49.4
19	12	5	950	1.5	6.84	54.2
11	13	5	900	1.5	6.79	53.5
20	14	5	950	1.5	6.84	54.2
10	15	7.5	950	1.5	6.82	50.7
5	16	2.5	900	2	6.79	53.6
15	17	5	950	1.5	6.84	54.2
9	18	2.5	950	1.5	6.85	54.8
12	19	5	1000	1.5	6.77	53.1
18	20	5	950	1.5	6.84	54.2
7	1	2.5	1000	2	6.765	52.4

#### 3.4.2 Statistical study of density.

The ANOVA results for the density response are presented in Table 4, demonstrating the significance of the developed quadratic model and its constituent terms. In general, a p-value less than 0.05 indicates that the corresponding model term significantly affects the response. The model exhibited a high F-value of 61.68 and a p-value of < 0.0001, confirming its statistical significance. Among the linear terms, composition (A), temperature (B), and holding time (C) were found to significantly influence density, with p-values of <0.0001, 0.0035, and 0.0035, respectively. Composition (A) exhibited the highest F-value (38.65), indicating that it is the most dominant factor governing the density response. In contrast, temperature and holding time contributed comparably, each possessing an F-value of 14.49. The interaction between composition and temperature (AB) was also statistically significant (p = 0.0175), while the AC and BC interactions were insignificant. Among the quadratic terms, B^2^ and C^2^ were significant, whereas A^2^ was statistically insignificant. Furthermore, the lack-of-fit was found to be insignificant (p = 0.3100), which is desirable and confirms that the developed model adequately represents the experimental data. Therefore, the proposed model is suitable for predicting the density response within the experimentally investigated domain.

**Table 4:** ANOVA for the prediction of density.

Source	∑Squares	d_f_	Mean Square	F-value	p-value	
Model	0.0345	9	0.00383	61.68	< 0.0001	**Significant**
A-Composition	0.0024	1	0.0024	38.65	< 0.0001	
B-Temperature	0.0009	1	0.0009	14.49	0.0035	
C-Holding time	0.0009	1	0.0009	14.49	0.0035	
AB	0.0005	1	0.0005	8.05	0.0175	
AC	3.125E-06	1	3.125E-06	0.05	0.8200	
BC	3.125E-06	1	3.125E-06	0.05	0.8200	
A²	0.0002	1	0.0002	3.22	0.1020	
B²	0.00089	1	0.00089	14.33	0.0037	
C²	0.0020	1	0.0020	32.20	< 0.0001	
Residual	0.00062	10	0.000062			
Lack of Fit	0.00040	5	0.00008	1.33	0.3100	**not significant**
Pure Error	0.00022	5	0.000044			

Table 5 confirms the quadratic nature of the model. The quadratic model exhibited a strong correlation with experimental data, as evidenced by R^2^ and adjusted R^2^ values of 99.90% and 99.40%, respectively, indicating that the developed quadratic model explains approximately 99.9% of the observed variation in density within the investigated experimental domain. These high R^2^ and adjusted R^2^ values reflect a strong correlation between the actual and predicted density values. However, the exceptionally high values of R^2^ and adjusted R^2^ for the model are considered appropriate given the present experimental conditions. One factor contributing to such a good fit of the data is the relatively small range of the design space utilized for the experiment using the CCD approach. Adequacy of the proposed model was also verified using R^2^ predictions, lack-of-fit analysis, and residual evaluation.

**Table 5:** Summary of Statistics model: Density.

Source	Std. Dev.	R²	Pred. R²	Adj. R²	PRESS	
Linear	04.35*10^−2^	0.1219	−0.0427	−0.5364	0.0530	
2FI	4.83*10^−2^	0.1229	−0.2819	−5.1071	0.2108	
Quadratic	1.8*10 ^**−3**^	**0.9990**	**0.9981**	**0.9940**	**0.0002**	**Suggested**
Cubic	2.2*10^−3^	0.9992	0.9974	−0.0255	0.0354	

Equation (1) represents the regression expression formulated to determine the density of composites. The second-degree polynomial includes all the input factors and their interactions, including square and multiplication terms.


$$\begin{array}{c} Density=+\text{6}.\text{8}\text{4}-\text{0}.\text{0}\text{1}\text{5}\text{5}A-\text{0}.\text{0}\text{0}\text{9}\text{5}B+\text{0}.\text{0}\text{0}\text{9}\text{5}C+\text{0}.\text{0}\text{0}\text{1}\text{9}AB+\text{0}.\text{0}\text{0}\text{0}\text{6}AC\;-\text{0}.\text{0}\text{0}\text{0}\text{6}BC-\text{0}.\text{0}\text{0}\text{2}\text{0}A^{\text{2}}-\text{0}.\text{0}\text{5}\text{7}\text{0}B^{\text{2}}-\text{0}.\text{0}\text{2}\text{7}\text{0}C^{\text{2}} \end{array}$$ (1)

(Note – A: Composition w.% graphite, B: Sintering temperature and C: Holding time)

**Fig 8:**
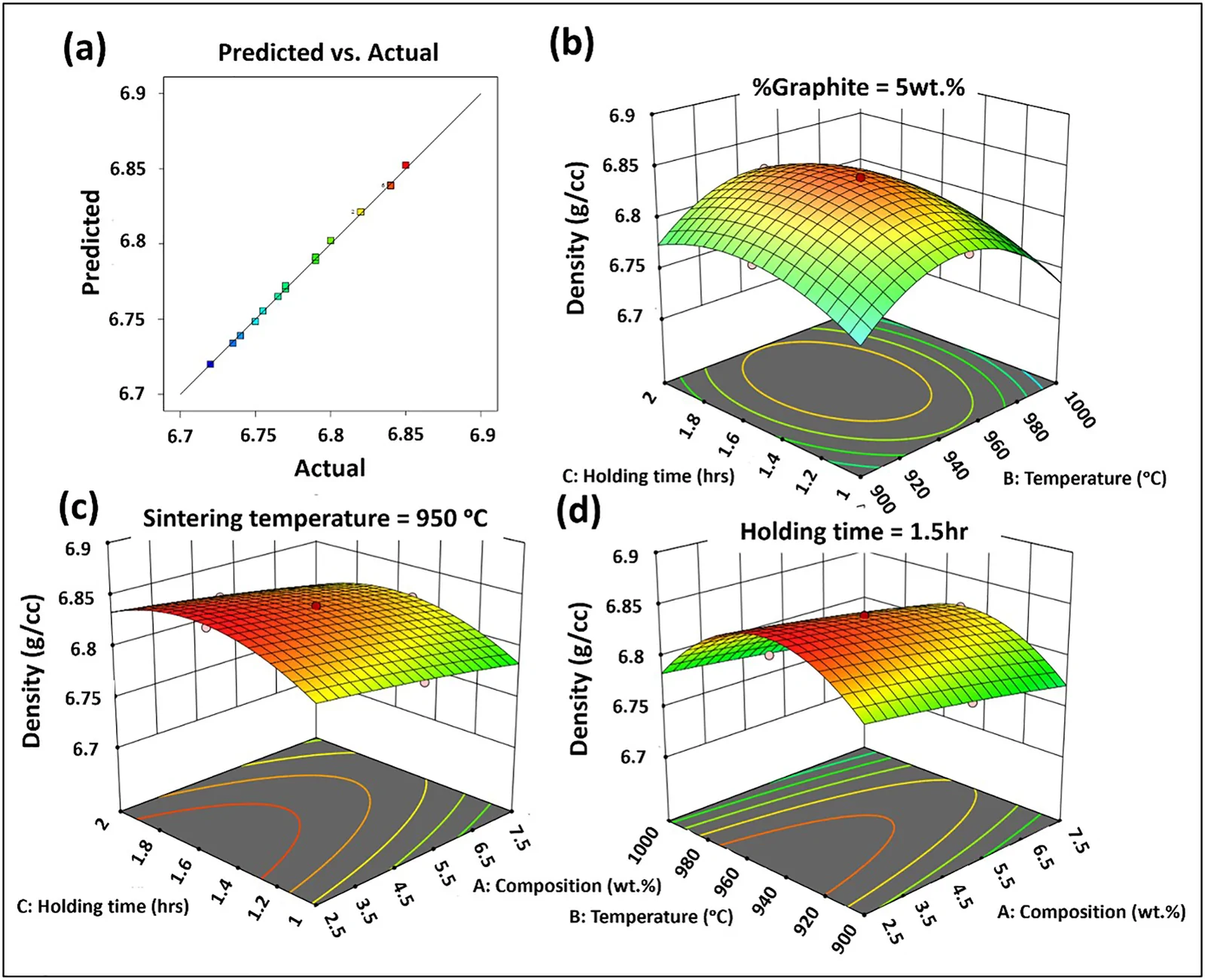
(a) Variation of predicted vs actual values of density, (b) interaction impact of temperature and holding time on density, (c) interaction impact of holding time and composition wt.% graphite on density and (d) interaction impact of temperature and composition on density.

Fig 8(a) presents the deviation between the predicted and experimental density measurements of the composite. The minor scatter around the straight line shows a strong relationship between the predicted and actual density values, with small error. The values show a good fit and are uniformly distributed along the line. The 3D contour plots in Fig 8(b)–8(d) represent the combined effects of the input parameters on composite density. Dots shown in the contour plots represent the design points used in the experimental matrix. The red dots denote data points that lie above the predicted values, whereas the white dots indicate points that fall below the predicted values. Also, the red color of the plots represents the highest density values, and the blue color represents the lowest density values for those specific input parameters. Fig 8(b) depicts the combined effect of temperature and holding time on density when graphite reinforcement is fixed at 5 wt.%. It is inferred that density increases with temperature and holding time, then decreases as the number of grains in the copper matrix increases. To analyze the combined effect of holding time and composition on density, the sintering temperature was held constant at 950°C, as shown in Fig 8(c). It is observed that density decreases as the graphite reinforcement weight percentage increases, owing to graphite’s lower density relative to copper. Also, to determine the combined influence of temperature and composition on density, holding time was held constant at 1.5 h, as shown in Fig 8(d). Maximum density is observed at a sintering temperature of 950 °C, and density decreases with composition owing to poor wetting between the graphite and the matrix.

**Fig 9:**
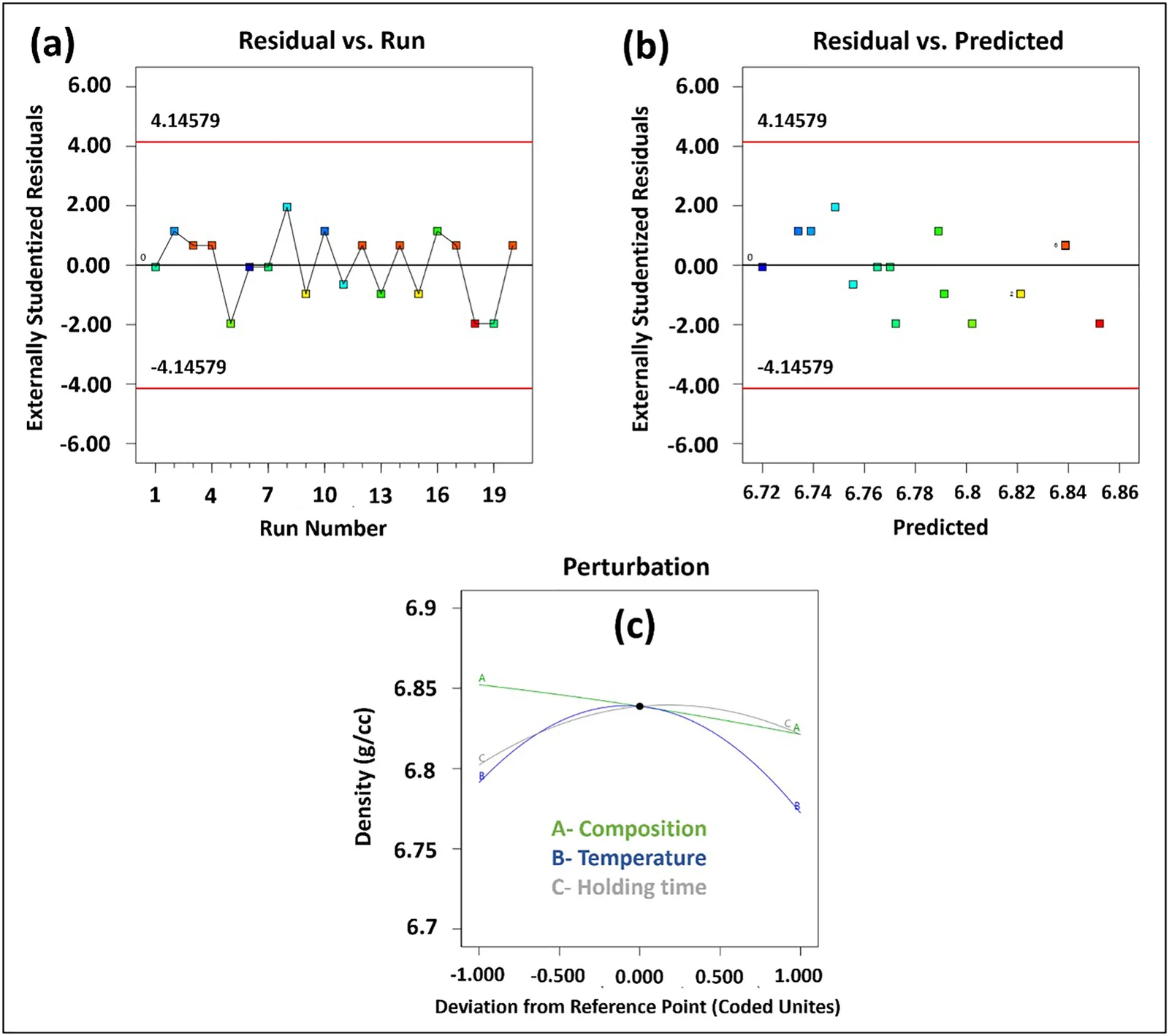
(a) Residual vs Run plot, (b) Residual vs Predicted plot and (c) Effect of individual factors on density.

Fig 9(a) shows the residual vs run plot, which shows a sequence of positive and negative values. It signifies that residuals are uniformly distributed. The residuals-versus-predicted plot is shown in Fig 9(b). The x-axis represents the model’s predictions, while the y-axis represents the residuals, indicating the deviation between the predicted and actual values. A positive value implies the prediction is too low, and a negative value implies the prediction is too high. These residual plots were examined to assess the adequacy of the developed regression models and to identify any potential overfitting. The residuals were found to be randomly distributed around the zero line, with no discernible systematic trends, indicating satisfactory model adequacy. The exceptionally high R^2^ values are considered to result from the relatively narrow and well-controlled experimental design space investigated in the present work rather than model over-parameterization. Nevertheless, the developed model should be applied only within the investigated ranges of graphite content, sintering temperature, and holding time. The influence of individual input factors on the composite density is determined using a perturbation plot, as shown in Fig 9(c). The perturbation plot was generated using a reference point corresponding to the central levels of all individual parameters: composition (graphite content: 5 wt.%), Temperature (950°C), and Holding time (1.5 h). The plot indicates that the density of the fabricated composites decreases with increasing reinforcement content, whereas it initially increases with temperature and holding time. After reaching the optimum, it starts decreasing with both holding time and temperature.

#### 3.4.3 Statistical study of hardness.

Table 6 presents the ANOVA results for hardness, illustrating the influence of the processing parameters on the response. The developed quadratic model was highly significant, as evidenced by an F-value of 1320 and a p-value < 0.0001. Among the linear terms, composition (A) and temperature (B) significantly affected hardness (p < 0.0001), whereas the linear effect of holding time (C) was statistically insignificant (p = 0.5170). However, the quadratic term C^2^ and the BC interaction term were statistically significant, indicating that the effect of holding time on hardness is nonlinear and dependent on its interaction with temperature. Composition (A) exhibited the highest F-value (6152.11), demonstrating that it is the most influential factor governing hardness. In addition, the lack-of-fit was found to be insignificant (p = 0.1350), confirming the adequacy of the developed model and its suitability for predicting the hardness response.

**Table 6:** ANOVA for the prediction of hardness.

Source	∑Squares	d_f_	Mean Square	F-value	p-value	
Model	84.31	9	9.37	1320.00	< 0.0001	**Significant**
A-Composition	43.68	1	43.68	6152.11	< 0.0001	
B-Temperature	1.16	1	1.16	163.38	< 0.0001	
C-Holding time	0.0032	1	0.0032	0.45	0.5170	
AB	0.0450	1	0.0450	6.34	0.0300	
AC	0.0200	1	0.0200	2.82	0.1240	
BC	0.2450	1	0.2450	34.51	0.0002	
A²	5.81	1	5.81	818.31	< 0.0001	
B²	2.25	1	2.25	316.90	< 0.0001	
C²	2.20	1	2.20	309.86	< 0.0001	
Residual	0.0710	10	0.0071			
Lack of Fit	0.0600	5	0.0120	2.40	0.1350	**not significant**
Pure Error	0.0110	5	0.0022			

While it is true that the model has high R^2^ values, their interpretation must be done in conjunction with other statistical measures. Based on the predicted R^2^, adjusted R^2^, lack-of-fit significance, and residual analysis, the model appropriately represents the experimental data in the design space. However, the regression equation developed can only be used to predict the response within the experimental domain; such values may indicate potential overfitting, particularly within a limited experimental design space. Therefore, the adjusted R^2^ and lack-of-fit tests are also used to assess the model’s adequacy and reliability.

Additionally, Table 7 indicates that a second-order model is suitable; it demonstrated a very high goodness of fit to the experimental data, with an R^2^ of 0.9992 and an adjusted R^2^ of 0.9925. These values indicate excellent agreement between the predicted and experimental responses within the investigated design space. A regression equation (quadratic) is also determined for the calculation of hardness as given in Eq. 2. The equation can be employed to estimate the hardness of the composite for any specified set of input parameters.

**Table 7:** Summary of Statistics model: Hardness.

Source	Std. Dev.	R²	Pred. R²	Adj. R²	PRESS	
Linear	1.57	0.5314	0.4435	0.1530	71.47	
2FI	1.74	0.5351	0.3205	−2.5618	300.56	
Quadratic	8.43*10 ^**−2**^	**0.9992**	**0.9984**	**0.9925**	**0.6291**	**Suggested**
Cubic	2.7*10^−3^	1.0000	1.0000	0.9994	0.0536	


$$\begin{array}{c} Hardness=+\text{5}\text{4}.\text{2}\text{0}-\text{2}.\text{0}\text{9}A-\text{0}.\text{3}\text{4}\text{0}\text{0}B+\text{0}.\text{0}\text{1}\text{8}\text{0}C+\text{0}.\text{0}\text{7}\text{5}\text{0}AB+\text{0}.\text{0}\text{5}\text{0}\text{0}AC-\text{0}.\text{1}\text{7}\text{5}\text{0}BC-\text{1}.\text{4}\text{5}A^{\text{2}}-\text{0}.\text{9}\text{0}\text{3}\text{6}B^{\text{2}}-\text{0}.\text{8}\text{9}\text{3}\text{6}C^{\text{2}} \end{array}$$ (2)

(Note – A: Composition w.% graphite, B: Sintering temperature and C: Holding time)

**Fig 10:**
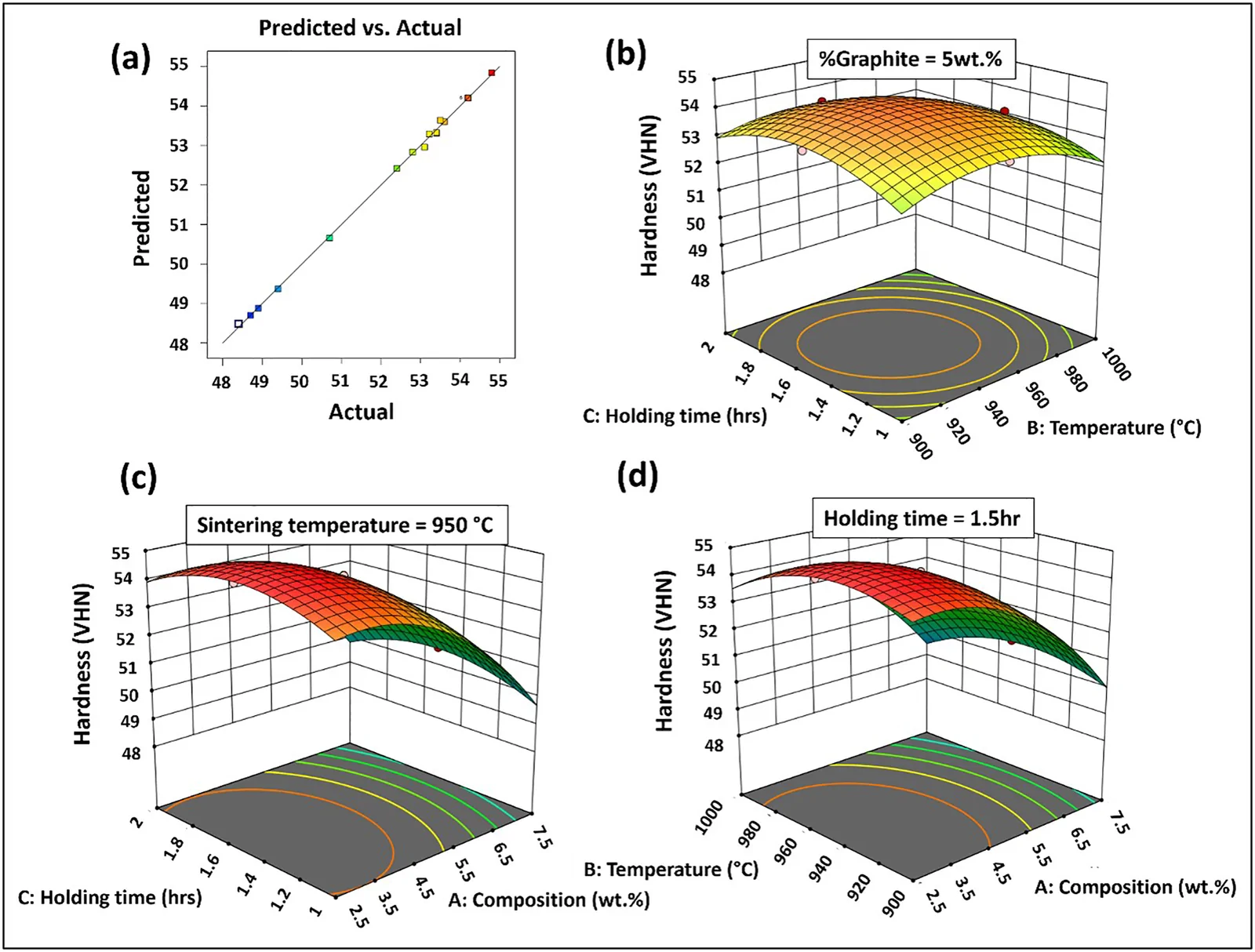
(a) Variation of predicted vs actual values of hardness of composite, (b) interaction impact of temperature and holding time on hardness, (c) interaction impact of holding time and composition wt.% graphite on hardness and (d) interaction impact of temperature and composition on hardness.

Fig 10(a) displays the variation between the predicted and actual values of the hardness of the composite. The deviation between the predicted and experimental values is marginal, suggesting good model accuracy [36,37]. Fig 10(b)–10(d) illustrate the 3D contour plots, which show the interactions between the independent factors and their combined impact on the hardness values. Red dots denote design points that lie above the predicted values, while the white dots designate points that fall below the predicted values. Fig 10(b) depicts the effect of temperature and holding time on hardness, with the graphite content held constant at 5 wt.%. The hardness increases with temperature and time, which may indicate enhanced interparticle bonding. After that, hardness decreases with a further increase in temperature and time, owing to the enlargement of matrix grains. Fig 10(c) demonstrates how graphite content and holding time jointly influence hardness at a sintering temperature of 950°C. From the 3D contour plot, it is seen that graphite content significantly reduces the hardness of the composite. The interaction between sintering temperature and composition on hardness is shown in Fig 10(d), with the holding time being constant at 1.5 h. Hardness increases with temperature up to 950°C due to strong bonding among powder particles; however, a slight decrease in hardness is observed at higher temperatures due to grain coarsening.

**Fig 11:**
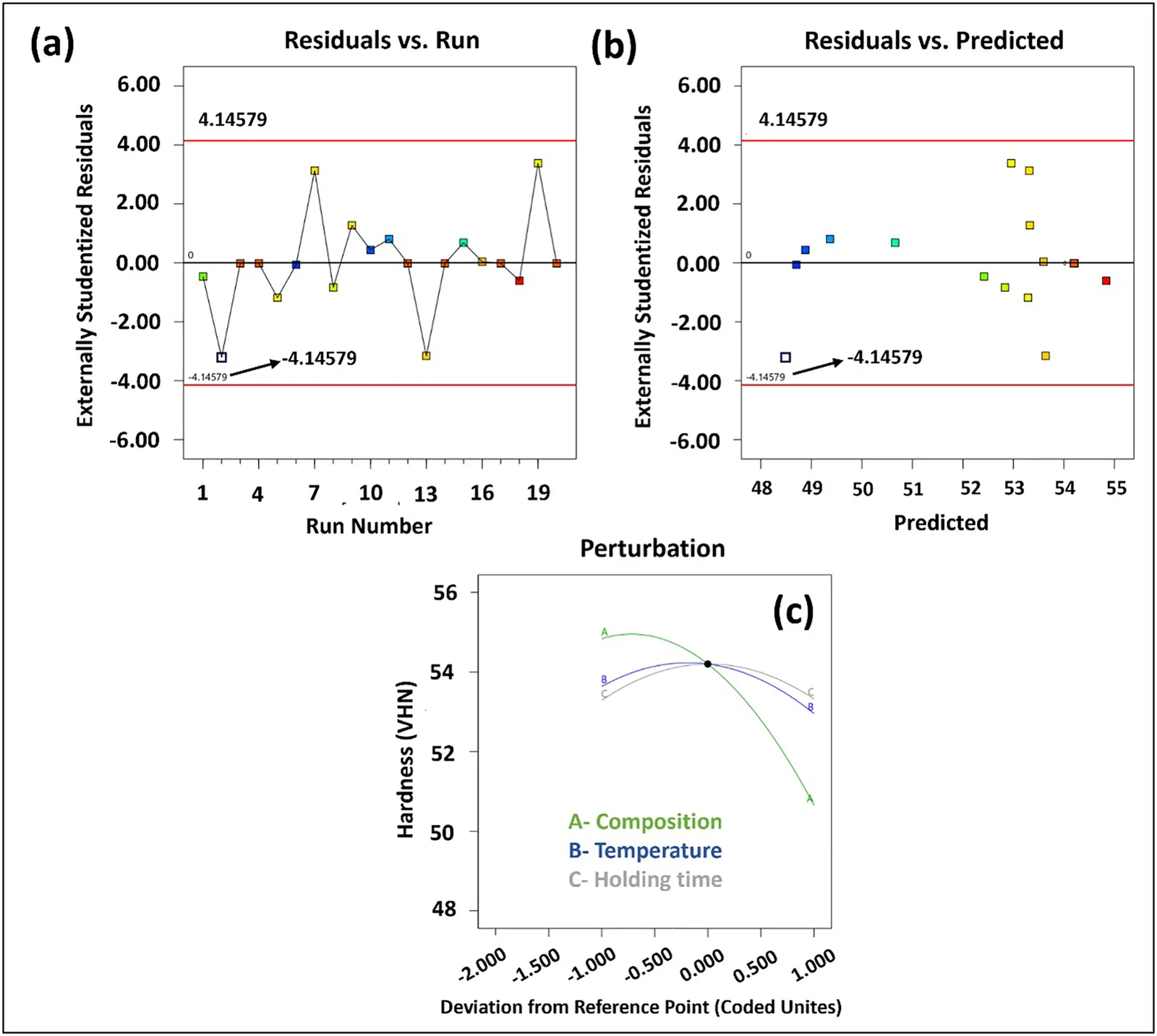
(a) Residual vs Run plot, (b) Residual vs Predicted plot and (c) Effect of individual factors on hardness.

The residuals versus run plot in Fig 11(a) shows that the residuals are randomly distributed within the allowable range, confirming the model’s adequacy. The residual vs predicted graph is depicted in Fig 9(b). The residual plots show a random distribution of data points, with most residuals clustered around the zero line, indicating a satisfactory model fit. A perturbation plot was produced using a reference point set at the central levels of individual factors-composition (graphite content- 5wt.%), Temperature (950°C), and Holding time (1.5 h), as revealed in Fig 11. The plot shows that the hardness of the fabricated composites decreases as the wt.% of graphite reinforcement is raised. In contrast, hardness initially increases with increasing sintering temperature and holding time, but after reaching an optimal point, it begins to decline as both parameters continue to increase.

#### 3.4.4 Validation of the developed model.

**Fig 12:**
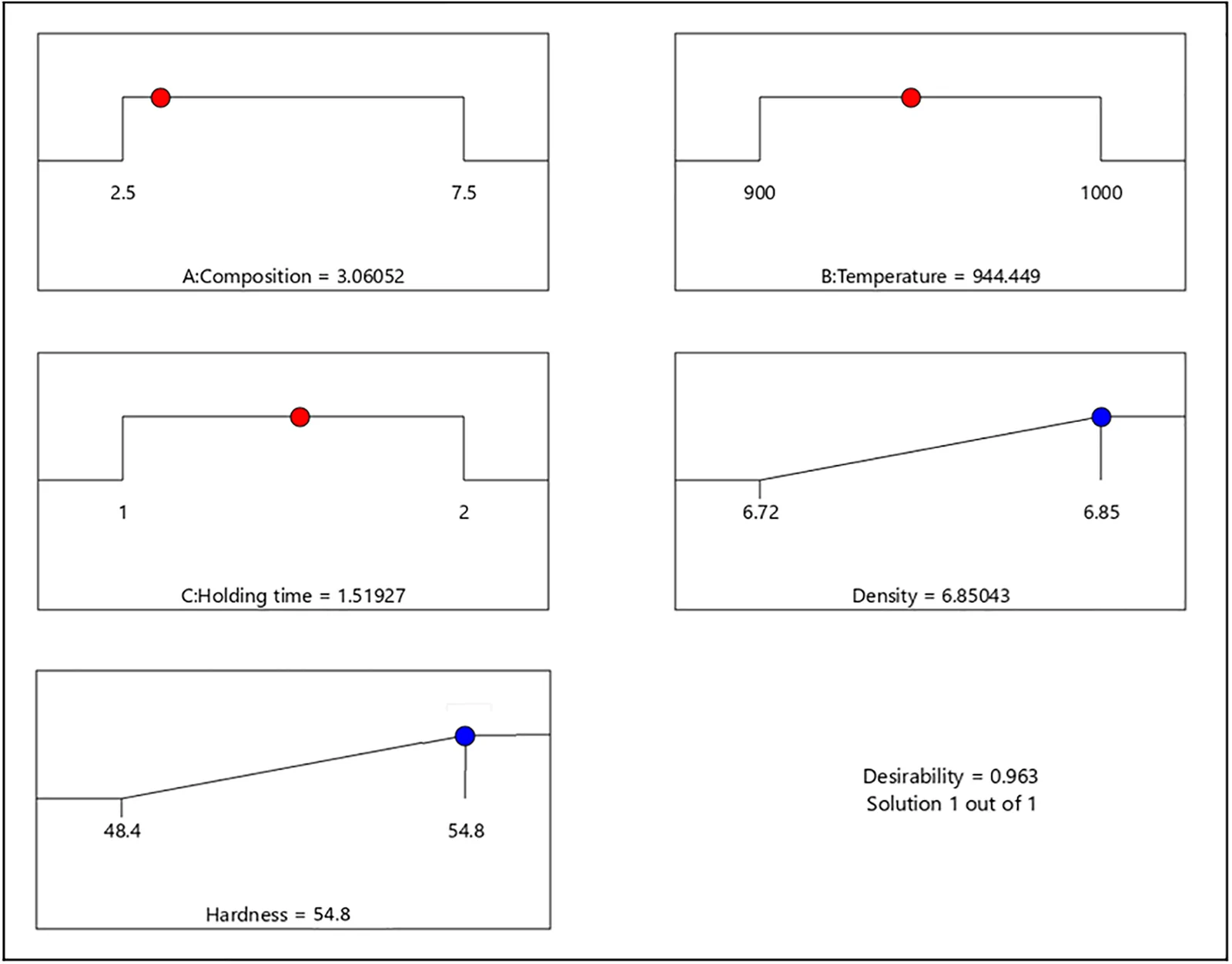
Predicted optimized outcomes of density and hardness by the model.

Fig 12 indicates that the maximum density and hardness achieved by the composites were approximately 6.85 g/cm^3^ and 54.8 VHN, respectively. Maximum density and hardness are attained when the input parameters are set to: composition (A) at 3.06 wt.% graphite, Temperature (B) at 944°C, and Holding time (C) at 1.52 h. To validate the developed model, a composite was fabricated using 3.06 wt.% graphite, a sintering temperature of 944 °C, and a holding time of 1.52 h, yielding experimentally obtained density and hardness values of 6.84 ± 0.05 g/cm³ and 54.5 ± 1.5 VHN, respectively. The differences between the experimental and predicted results for density and hardness were approximately 0.14% and 0.54%, respectively.

Thus, the model demonstrates good predictive capability within the studied parameter range, as confirmed by the close agreement between predicted and experimental results. The model is reliable for effectively predicting the density and hardness of Cu-Gr composites. The validation performed in this study is based on confirmation experiments within the design space. However, the absence of independent external or cross-validation may limit the model’s generalizability. It is also important to note that the predictive models obtained in the present work are valid within the investigated ranges of graphite concentration, sintering temperature, and holding time. Even though confirmation tests showed good agreement between predicted and experimental data, the external validation of the models was not done. Thus, any extrapolation beyond the investigated range of processing parameters should be made with caution.

## 4. Conclusions

In the present study, graphite-reinforced copper composites were successfully manufactured using the powder metallurgy route by varying the graphite content, sintering temperature, and sintering (holding) time. A comprehensive study was performed using RSM and ANOVA to analyze the most important parameters affecting the density and hardness of the prepared composites. Regression models were developed to predict density and hardness as functions of specific input parameters. The major conclusions drawn from the study are as follows:

1. An increase in graphite content in Cu–Gr composites resulted in a measurable reduction in both density and hardness. Specifically, increasing graphite from 2.5 wt.% to 7.5 wt.% led to decreases of 0.44% in density and 7.07% in hardness, indicating a strong compositional influence on mechanical performance.
2. RSM identified the optimal processing parameters within the investigated range for maximizing density and hardness as 3.06 wt.% graphite, 944 °C sintering temperature, and 1.52 h holding time, with composition emerging as the dominant factor. The developed regression models showed high predictive accuracy, with only 0.14% error for density and 0.54% error for hardness between experimental and predicted values.
3. These results confirm that RSM is an efficient and reliable prediction tool for property prediction and process parameter tuning. The approach offers significant potential for industrial-scale composite manufacturing by reducing trial-and-error experimentation, minimizing resource consumption, and enabling data-driven material design for future high-performance applications.
4. Future work should focus on validating the model using independent datasets to establish its robustness further.
