# Supplementary material for: Prediction of the properties of sintered copper-graphite composites using response surface methodology
Source: PLoS One. 2026 Sep 11;21(9):e0357759. doi: 10.1371/journal.pone.0357759 (PMC13567752; doi:10.1371/journal.pone.0357759)
Supplement: Supplementary file 1 [file pone.0357759.s001.docx]

**General Editorial Questions**

**Comment 1: Is the manuscript technically sound, and do the data support the conclusions?**
Reviewer #1: Partly

Reviewer #2: Partly

**Response:** The manuscript has been revised to improve technical clarity and strengthen the linkage between experimental data and conclusions. Additional explanations have been incorporated to better justify observed trends, and limitations of the study have now been explicitly acknowledged. While the experimental framework remains valid, the discussion has been expanded to avoid overgeneralization and to ensure that conclusions are fully supported by the presented data.

**Comment 2: Has the statistical analysis been performed appropriately and rigorously?**

Reviewer #1: No

Reviewer #2: Yes

**Response:** We appreciate the reviewers’ observations. The statistical analysis has been carefully re-evaluated. Errors and inconsistencies in the ANOVA tables have been corrected, and additional statistical validation—such as residual analysis and diagnostic plots—has now been included. The interpretation of statistical parameters has also been revised to align with standard practices.

**Comment 3: Have the authors made all data underlying the findings fully available?**

Reviewer #1: Yes

Reviewer #2: Yes

**Response:** All relevant data supporting the findings are included within the manuscript. In addition, structured datasets and supporting numerical values have now been organized more clearly.

**Comment 4: Is the manuscript written in standard English and presented clearly?**

Reviewer #1: No

Reviewer #2: No

**Response:** The manuscript has undergone thorough language editing to improve clarity, grammar, and readability. Redundant statements have been removed, and the overall structure has been refined to ensure clear and concise scientific communication.

**Reviewer #1:**

The present manuscript addresses a topic of industrial relevance (Cu-Gr composites); however, it fails to provide a significant scientific contribution or a detailed mechanistic investigation. There is a lack of robust discussion regarding sintering physics, diffusion kinetics at the Cu-Gr interface, or the influence of graphite morphology on the strain hardening of the matrix. The study is incremental and lacks the analytical rigor required by high-impact journals. While the study is presented as an investigation into “predictive modelling” and “sustainable manufacturing,” it is, in practice, reduced to a quadratic Response Surface Methodology (RSM) fit over a restricted experimental set. The work suffers from insufficiently defined variables, statistical inconsistencies in the ANOVA, unsubstantiated microstructural interpretations, and an inadequate formulation of density as a response variable when comparing distinct compositions. In particular, using absolute density as an optimization parameter while varying graphite content introduces an obvious conceptual bias: the reduction in theoretical density with increasing graphite fraction stems directly from the Rule of Mixtures, regardless of sintering efficiency. Therefore, it is indispensable to work with relative density or explicit porosity metrics. Furthermore, central conclusions, such as the existence of a precise processing optimum and clear trends in density and hardness regarding temperature and time, are not supported by the necessary statistical robustness, as the observed variations are minor and lack proper experimental uncertainty analysis.
Microstructural evidence is also insufficient to support categorical claims of "homogeneous dispersion," as the presented micrographs and EDS maps are qualitative, limited in scope, and lack spatial quantification. Specifically, regarding EDS carbon mapping in Cu–C composites, the physical limitations of the technique demand a much more cautious interpretation. Similarly, the XRD analysis has been extrapolated beyond its capabilities: the absence of additional peaks does not permit the conclusion that there is no reaction between copper, graphite, and oxygen; it merely indicates the absence of phases above the detection limit under specific test conditions. Without Rietveld refinement or a discussion on detection limits across multiple sintering conditions, such a generalization is improper.

In summary, the manuscript remains essentially incremental, confirming expected trends for powder metallurgy Cu–Gr composites without offering a sufficient experimental or physical basis to sustain claims of predictive optimization or industrial-scale sustainable manufacturing.

**Major´s**
**Comment** 1. Why did the authors choose absolute density as a response variable while simultaneously varying the composition?

**Response:** Absolute density was selected as a response variable to directly quantify the extent of densification and porosity evolution during sintering, enabling a consistent comparison of microstructural consolidation despite variations in composite composition.

**Comment** 2. Are Eqs. (1) and (2) written in actual (real) or coded variables?

**Response:** The equations are in coded variables

**Comment** 3. How do the authors explain the internal inconsistencies found in the ANOVA tables?

**Response:** The authors sincerely apologize for the inconsistencies identified in the ANOVA table. Upon careful re-examination, it was found that the discrepancies were due to errors in data transcription and calculation. To address this, the ANOVA analysis has been completely re-performed, and all statistical parameters (sum of squares, mean squares, F-values, and p-values) have been recalculated to ensure internal consistency and accuracy. The corrected ANOVA table has now been incorporated into the revised manuscript.

**Comment** 4. Why do the authors interpret the p-value as “99% confidence” or “99% probability of correct prediction”?

**Response:** The authors acknowledge that interpreting the p-value as ‘99% confidence’ or ‘probability of correct prediction’ is not statistically rigorous. Although similar interpretations have been reported in prior studies, the manuscript has been revised to adopt a statistically correct interpretation, where p-values are used to indicate the significance of model terms rather than predictive accuracy. (references: S.C. Vettivel et al, Numerical modelling, prediction of Cu–W nano powder composite in dry sliding wear condition using response surface methodology, Materials and Design 50 (2013) 977–996), Md Syam Hasan et al, Analysis of the friction and wear of graphene reinforced aluminum metal matrix composites using machine learning models, Tribology International 170 (2022) 107527

**Comment** 5. What specific microstructural evidence supports the claims of grain coarsening, increased pore size, and decreased wettability?

**Response:** The microstructural claims are supported by both optical microscopy and SEM analysis. The optical micrographs (Fig. 3) reveal grain morphology, where an increase in grain size at higher sintering temperatures indicates grain coarsening. Additionally, SEM images provide clearer evidence of pore characteristics, showing enlargement and increased pore connectivity at elevated temperatures. The presence of interfacial gaps and non-uniform bonding between graphite and copper in SEM micrographs, further supported by elemental mapping, indicates reduced wettability between the reinforcement and matrix. The manuscript has been revised to explicitly highlight these observations.

**Comment** 6. On what basis do the authors claim “uniform dispersion” of graphite?

**Response:** The claim of graphite dispersion is based on SEM micrographs and corresponding elemental mapping, which indicate a largely homogeneous distribution of graphite within the copper matrix. However, careful observation also reveals minor agglomeration in some regions, likely due to the finer particle size of graphite. Accordingly, the manuscript has been revised to reflect a more accurate description as “mostly uniform dispersion” rather than complete uniformity, along with the supporting microstructural evidence.

**Comment** 7. Why was XRD used to definitively assert the absence of reaction between copper, graphite, and oxygen?

**Response:** XRD was employed to identify any possible phase formation resulting from reactions among copper, graphite, and oxygen. The diffraction patterns show only the characteristic peaks of copper and graphite, with no additional peaks corresponding to oxide phases (such as CuO or Cu₂O) or interfacial reaction products. This absence of new phases within the detection limit of XRD suggests that no significant chemical reaction occurred during processing.

**Comment** 8. How do the authors validate a “predictive model” without independent external validation?

**Response:** The predictive capability of the developed model was validated through confirmation experiments using optimized input parameters obtained from the model. The composite was fabricated at these conditions, and the experimentally measured values of density and hardness were compared with the predicted values. The close agreement between experimental and predicted results, with errors of 0.14% for density and 0.54% for hardness, demonstrates the adequacy and reliability of the model within the studied parameter range. The manuscript has been revised to clarify that this validation is based on confirmation experiments rather than independent external validation.

**Comment** 9. Is the reported uncertainty for experimental hardness at the optimum point realistic? (The manuscript reports 54.5 ± 0.0015 VHN).

**Response:** The authors acknowledge their fault and correction has been made in the manuscript.

**Comment** 10. How did the authors rule out powder contamination during milling with WC balls?
**Response:** The milling of copper and graphite powders was performed in a tungsten carbide vial and with WC balls, that are much softer than tungsten carbide. So, any contamination by balls assumed to be negligible.

**Comment** 11. What is the functional relevance of the study for applications such as brushes and pantographs if the manuscript does not measure electrical conductivity or tribological properties?

**Response:** The authors acknowledge that electrical conductivity and tribological performance are critical for applications such as brushes and pantographs. The present study is focused on establishing the processing–microstructure–mechanical property relationship (density and hardness) as a foundational step. These properties are essential for ensuring structural integrity and wear resistance, which are prerequisites for such applications. However, detailed evaluation of electrical and tribological properties will be addressed in future work.

**Comment** 12. In what specific sense does this work constitute “sustainable manufacturing”?

**Response:** The authors consider this work aligned with sustainable manufacturing in the context of process optimization. The development of a regression-based predictive model reduces the need for extensive trial-and-error experimentation, thereby minimizing material usage, energy consumption, and processing time. This contributes to resource efficiency and waste reduction during composite fabrication. The manuscript has been revised to clarify this aspect more explicitly.

**Comment** 13. Why is the experimental design space so restricted to support a strong conclusion of a global optimum?

**Response:** The authors acknowledge that the experimental design space is limited to the selected ranges of composition, sintering temperature, and holding time. Therefore, the reported optimum corresponds to the optimal conditions within the investigated parameter domain, rather than a global optimum. The chosen ranges were based on preliminary trials and relevant literature to ensure practical feasibility. The manuscript has been revised to clarify that the optimization results are valid within the studied design space**.**

**Comment** 14. Can the authors provide green density, linear shrinkage after sintering, and apparent/closed porosity for each condition? Without these data, the densification discussion remains incomplete.

**Response:** The authors acknowledge that parameters such as linear shrinkage and porosity are important for a comprehensive understanding of densification behaviour. In the present study, green density and sintered density have been reported to evaluate densification trends. However, linear shrinkage and apparent/closed porosity were not measured during experimentation. The manuscript has been revised to clarify this limitation, and these aspects will be considered in future work to provide a more complete densification analysis.

**Comment** 15. What was the criterion for choosing a 0.5 kg load and 30 s dwell time for microhardness, considering the composite's heterogeneity? Did the authors verify if indentations intercepted distinct regions of Cu, graphite, and pores?

**Response:** The load of 0.5 kg (HV0.5) and dwell time of 30 s were selected based on standard microhardness testing practices for metal matrix composites, ensuring a measurable and well-defined indentation while minimizing excessive influence from localized heterogeneities. To account for microstructural non-uniformity, multiple indentations were performed at different locations on each sample, and the reported hardness values represent the average of these measurements. Care was taken to avoid placing indentations directly on visible pores or large graphite clusters. However, given the heterogeneous nature of Cu–Gr composites, some degree of phase interaction within the indentation zone is unavoidable. This averaging approach ensures that the reported hardness values are representative of the overall composite behaviour rather than individual phases.

**Comment 16.** Why was no residual analysis of the model presented? Where are the normal probability plots, residuals vs. predicted, residuals vs. run order, leverage/influence, and outlier diagnostics? A quadratic model with such a high R2 requires a statistical audit, not automatic celebration.

**Response:** The authors have added the adequate plots for both density (Fig.8) and hardness (Fig.11) of composite.

**Comment 17.** Why does composition appear as the dominant factor for both density and hardness, yet the authors fail to clearly separate the intrinsic effect of composition from the processing effect? Currently, the model conflates both and attributes the result to “optimization of sintering parameters,” which is conceptually imprecise.

**Response:** The authors thank the reviewer for this observation and apologize for the lack of clarity. To address this concern, the title has been revised to more accurately reflect the scope of the study and avoid any ambiguity between compositional and processing effects.

**Reviewer #2:**

The manuscript presents an experimental and statistical investigation on the optimization of Cu–graphite composites fabricated via powder metallurgy using Response Surface Methodology (RSM) and ANOVA. The study is relevant for materials engineering and sustainable manufacturing, and the methodology employed is generally appropriate. The experimental design (CCD), statistical modelling, and validation approach are sound, and the results are consistent with expected physical behavior.
 However, the manuscript requires major revision before it can be considered for publication. The main concerns are outlined below:

**Major Comments**

**Comment 1: Language and Clarity**

The manuscript requires substantial improvement in English language and scientific writing. There are numerous grammatical errors, awkward sentence constructions, and unclear expressions throughout the text. Additionally, there is noticeable redundancy in the abstract, where portions of text are repeated. A thorough professional language revision is strongly recommended.

**Response:** The manuscript has been thoroughly revised for grammar, clarity, and readability. Redundancies have been removed, and the abstract has been rewritten.

**Comment 2: Limited Scientific Novelty**: While the application of RSM and ANOVA is appropriate, the manuscript primarily presents an application of well-established statistical techniques. The novelty of the work is therefore limited. The authors are encouraged to better highlight the scientific contribution of the study, particularly in comparison with existing literature.

**Response:** The authors acknowledge the reviewer’s concern regarding novelty. The manuscript has been revised to more clearly highlight the scientific contribution, which lies in the integrated experimental–statistical framework applied specifically to Cu–Gr composites over a defined processing window. Unlike existing studies, this work establishes a quantitative correlation between composition, sintering parameters, and combined densification–hardness behaviour, along with validated optimization.

**Comment 3: Lack of Deeper Physical Interpretation:** The discussion section is largely descriptive. The authors should provide a more in-depth physical interpretation of the observed trends, particularly:

The trade-off between densification and grain growth at higher temperatures and holding times the role of graphite in reducing density and hardness the mechanisms governing the existence of an optimal point (~3 wt.% graphite, ~944°C, ~1.5 h)

Strengthening this discussion would significantly improve the scientific impact of the manuscript.

**Response:** The discussion has been expanded to include: Densification vs grain growth trade-off , Role of graphite in modifying density and hardness, Physical reasoning behind the observed optimum.

**Comment 4:** Statistical Analysis – Need for Critical Discussion

Although the statistical methodology is correctly applied, the manuscript lacks a critical assessment of the model robustness. In particular:

The very high R² values (≈ 0.999) should be discussed, as they may indicate potential overfitting

No external validation or cross-validation is presented

Including a discussion on model limitations would improve the reliability of the results.

**Response:** The authors thank the reviewer for highlighting this important aspect. The manuscript has been revised to include a critical discussion on model robustness. The very high R² values (≈ 0.999) are now discussed with respect to the possibility of overfitting, particularly considering the limited design space and number of experimental runs. To address this, additional statistical indicators such as adjusted R² and lack-of-fit analysis have been emphasized to demonstrate model adequacy. Furthermore, it is clarified that the model validation was performed through confirmation experiments rather than independent external validation. The absence of cross-validation or external datasets is now explicitly acknowledged as a limitation, and its impact on model generalizability has been discussed. These additions provide a more balanced and critical assessment of the developed model.

**Minor Comments**

**Abstract**
Remove duplicated sentences and improve clarity Make the abstract more concise and focused on key quantitative results

**Response:** The authors thank the reviewer for the valuable suggestion. The abstract has been revised to remove redundancy, improve clarity, and present key quantitative results in a more concise and focused manner.

**Data Availability**

The authors state that data are available within the manuscript; however, it would be beneficial to clearly provide raw data or structured datasets, preferably in a supplementary file or public repository, in line with PLOS data policies

**Response:** The authors acknowledge the importance of data transparency. The data presentation has been improved, and all the data are included in the manuscript.

**Terminology and Writing**

Replace informal or incorrect expressions (e.g., “fainted” → “decreased”) Improve consistency in terminology (e.g., “holding time” vs “sintering time”)

**Response:** The manuscript has been carefully revised to correct informal expressions and ensure consistency in technical terminology. Terms such as “holding time” and “sintering time” have been standardized throughout the manuscript.

**Figures and Discussion**

Some figures are well presented, but their interpretation could be more concise and insightful rather than purely descriptive

**Response:** The figure descriptions and associated discussion have been revised to be more concise and interpretation-focused, emphasizing key insights rather than purely descriptive content.

**Final Recommendation**

The study is technically sound and the results are meaningful; however, significant improvements in language, scientific depth, and discussion are required. Addressing the points above will substantially enhance the quality and impact of the manuscript.

**Response:** The authors sincerely thank the reviewer for the constructive feedback. Significant revisions have been made to improve the language quality, scientific depth, and discussion. These changes have substantially enhanced the clarity, rigor, and overall impact of the manuscript.
