# Supplementary material for: Prediction of the properties of sintered copper-graphite composites using response surface methodology
Source: PLoS One. 2026 Sep 11;21(9):e0357759. doi: 10.1371/journal.pone.0357759 (PMC13567752; doi:10.1371/journal.pone.0357759)
Supplement: Supplementary file 2 [file pone.0357759.s002.docx]

**RESPONSE SHEET**

**Journal Requirements**

When submitting your revision, we need you to address these additional requirements.

1. Please ensure that your manuscript meets PLOS ONE's style requirements, including those for file naming. The PLOS ONE style templates can be found at https://journals.plos.org/plosone/s/file?id=wjVg/PLOSOne_formatting_sample_main_body.pdf and https://journals.plos.org/plosone/s/file?id=ba62/PLOSOne_formatting_sample_title_authors_affiliations.pdf

**Response:** The manuscript has been structured according to PLOS ONE's style requirements.

2. Please note that PLOS One has specific guidelines on code sharing for submissions in which author-generated code underpins the findings in the manuscript. In these cases, we expect all author-generated code to be made available without restrictions upon publication of the work. Please review our guidelines at https://journals.plos.org/plosone/s/materials-and-software-sharing#loc-sharing-code and ensure that your code is shared in a way that follows best practice and facilitates reproducibility and reuse.

**Response:** The authors have complied according to the guidelines.

3. We note that Figure 1 in your submission contains images which may be copyrighted. All PLOS content is published under the Creative Commons Attribution License (CC BY 4.0), which means that the manuscript, images, and Supporting Information files will be freely available online, and any third party is permitted to access, download, copy, distribute, and use these materials in any way, even commercially, with proper attribution. For more information, see our copyright guidelines: http://journals.plos.org/plosone/s/licenses-and-copyright.

We require you to either (1) present written permission from the copyright holder to publish these figures specifically under the CC BY 4.0 license, or (2) remove the figures from your submission:

**Response:** Figure 1 of the manuscript contain images that are originally taken by the author. The authors have worked on the ball milling machine and furnace shown in the Figure 1.

**General Editorial Questions**

**Comment 1: Is the manuscript technically sound, and do the data support the conclusions?**
Reviewer #1: Partly

Reviewer #2: Partly

**Response:** The manuscript has been revised to improve technical clarity and strengthen the linkage between experimental data and conclusions. Additional explanations have been incorporated to better justify the observed trends, and limitations of the study have now been explicitly acknowledged. While the experimental framework remains valid, the discussion has been expanded to avoid overgeneralization and to ensure that conclusions are fully supported by the presented data.

**Comment 2: Has the statistical analysis been performed appropriately and rigorously?**

Reviewer #1: No

Reviewer #2: No

**Response:** We appreciate the reviewers’ observations. The statistical analysis has been carefully re-evaluated. Errors and inconsistencies in the ANOVA tables have been corrected, and additional statistical validation—such as residual analysis and diagnostic plots—has now been included. The interpretation of statistical parameters has also been revised to align with standard practices.

**Comment 3: Have the authors made all data underlying the findings fully available?**

Reviewer #1: No

Reviewer #2: Yes

**Response:** All relevant data supporting the findings are included within the manuscript. The experimental data of density and hardness are shown in Figures 4-6. In addition, structured datasets and supporting numerical values have now been organized more clearly (given in Table 3).

**Comment 4: Is the manuscript written in standard English and presented clearly?**

Reviewer #1: No

Reviewer #2: Yes

**Response:** The manuscript has undergone thorough language editing to improve clarity, grammar, and readability. Redundant statements have been removed, and the overall structure has been refined to ensure clear and concise scientific communication.

**Reviewer #1:**

I previously reviewed an earlier version of this manuscript, and although the revised submission shows noticeable improvements in organization, statistical presentation, and discussion of some physical mechanisms, several important concerns raised in the previous review remain insufficiently addressed. In my opinion, these unresolved issues are substantial and still justify a recommendation of major revision.

Important methodological and reproducibility concerns remain unresolved.

**Major´s**
**Comment** 1. Robustness and predictive reliability of the statistical model

The manuscript continues to report extremely high R² values (~0.999) obtained from a relatively limited experimental design space. Although the revised version now briefly acknowledges the possibility of overfitting, no independent external validation or cross-validation procedure has been performed. The validation remains restricted to confirmation experiments conducted very close to the optimized condition itself, which is not sufficient to fully demonstrate the true predictive capability or generalizability of the proposed model. Since the work strongly emphasizes predictive modelling and optimization, stronger validation procedures are necessary to support these claims

**Response:** We thank the reviewer for this important comment. In the present study, the models were developed using Response Surface Methodology (RSM) based on the experimental data generated within the investigated design space, and their adequacy was evaluated through analysis of variance (ANOVA), residual analysis and predicted-versus-actual plots. However, additional validation approaches, such as cross-validation, independent test datasets, or experiments conducted at new design points outside the optimization region, would strengthen confidence in the model's generalizability. These aspects are beyond the scope of the present study and will be considered in future work

**Comment** 2. Experimental reproducibility and data availability

The manuscript still states only that “Data is provided within the manuscript. ” However, no structured raw dataset, supplementary spreadsheet, or publicly accessible repository has been provided. Considering that the study relies heavily on ANOVA/RSM modelling and statistical optimization, the absence of openly accessible experimental datasets substantially limits reproducibility, transparency, and independent verification of the reported results. This remains an important concern, particularly under the reproducibility and data-sharing standards expected by PLOS ONE.

**Response:** The experimental data is provided in the Table 3 for both density and hardness of the composite.

**Comment** 3. Scientific contribution and scope

While the manuscript has improved its discussion and presentation, the core contribution still remains largely centered on the application of established statistical optimization techniques (RSM/ANOVA) to a relatively restricted processing window of Cu–Gr composites. The broader scientific impact and methodological novelty of the study could still be more clearly established.

**Response-** We thank the reviewer for this thoughtful assessment and constructive suggestion. The primary objective of the present study was not to develop a new statistical methodology but rather to systematically investigate the combined effects of graphite content, sintering temperature, and sintering time on the density and hardness of Cu–Gr composites and to establish empirical relationships between these processing variables and the resulting properties. The contribution of the work lies in providing a statistically guided optimization framework for Cu–Gr composites fabricated through powder metallurgy and in identifying the parameter combinations that maximize the measured responses within the investigated experimental domain. Further, the RSM technique can be used for other metal matrix composites, also for analysis of tribological performance the composites.

**Comment** 4. Language and scientific writing

Although improved compared with the previous version, the manuscript still contains several grammatical inconsistencies and awkward sentence constructions throughout the text. Additional professional language revision would improve clarity and readability.

the remaining concerns regarding validation robustness, predictive reliability, reproducibility, and data transparency are still significant and should be more thoroughly addressed before the manuscript can be considered for publication.

**Response-** The authors acknowledge the reviewer comment. The manuscript has undergone a thorough revision to improve grammar, sentence structure, clarity, and overall readability. The text has been carefully reviewed, and several grammatical inconsistencies have been corrected throughout the manuscript. With respect to the concerns related to validation robustness, predictive reliability, reproducibility, and data transparency, we acknowledge the importance of these aspects. The scope and limitations of the developed RSM models have been further clarified in the revised manuscript.

**Reviewer #2:**

The manuscript addresses an applied and industrially relevant topic and is currently in its second revision; however, in its present form, it still falls short of the scientific rigor required to support its central claims of “predictive modelling,” “optimization,” and “sustainable manufacturing.” The revision carried out by the authors has improved certain aspects of writing and transparency, but it has not resolved the core conceptual and methodological problems previously identified by the reviewers. The study remains essentially based on a quadratic RSM/CCD fit over a restricted experimental space, with a predominantly qualitative physical interpretation and internal statistical inconsistencies that are still present in the revised text. For example, one problematic issue remains the choice of absolute density as a response variable while the graphite content is simultaneously varied. The manuscript itself acknowledges that density decreases with increasing graphite content due to the intrinsic difference between the densities of copper and graphite, in addition to possible effects of wettability and agglomeration. This means that the “density” response is inevitably confounded by the rule of mixtures, without adequately isolating the actual densification efficiency. Under these conditions, the interpretation of the processing optimum becomes conceptually weakened. Furthermore, although the authors claim to have corrected the ANOVA and strengthened the statistical validation, the revised text still contains internal inconsistencies. In the density ANOVA, temperature and time appear with p = 0.0035, yet the text states that all three factors have p < 0.0001; in the hardness ANOVA, term C (holding time) appears with p = 0.5170, and is therefore not significant, but the text states that A, B, and C are significant with p < 0.0001. There is also inconsistency in the R² and adjusted R² values discussed for the density model. This compromises the reliability of the statistical analysis.

**Response:** The authors sincerely thank the reviewer for the careful and constructive assessment of the manuscript. We acknowledge that several statements in the previous version generalized the significance of model terms and inadvertently introduced inconsistencies between the ANOVA tables and their interpretation. The manuscript has been thoroughly revised to ensure complete consistency between the statistical outputs and the accompanying discussion.

For the density model, the previous statement indicating that all three factors possessed p-values lower than 0.0001 has been corrected. The revised text now accurately reports that composition (A), temperature (B), and holding time (C) are statistically significant with p-values of <0.0001, 0.0035, and 0.0035, respectively. Likewise, the values of R² and adjusted R² have been carefully verified and corrected wherever necessary. Similarly, for the hardness model, the previous interpretation has been revised to reflect the actual ANOVA results. The linear effect of holding time (C) was found to be statistically insignificant (p = 0.5170), and therefore the earlier statement suggesting that all three linear factors possessed p-values lower than 0.0001 has been removed. The revised discussion now clarifies that the effect of holding time on hardness is predominantly nonlinear, as evidenced by the highly significant quadratic term (C², p < 0.0001) and the significant BC interaction term (p = 0.0002). Consequently, holding time contributes to the hardness response through curvature and interaction effects rather than through a simple linear relationship.

In addition, the sentence referring to possible inconsistencies arising from rounding of numerical values has been removed to avoid ambiguity and to maintain the integrity of the statistical analysis. With regard to the reviewer's concern regarding the use of density as a response variable, the authors agree that the absolute density of Cu–graphite composites is inherently influenced by the density difference between copper and graphite according to the rule of mixtures. Therefore, a reduction in density with increasing graphite content cannot be interpreted solely as a reduction in densification efficiency. To address this concern, the discussion has been revised to emphasize that the measured density values represent the combined influence of composition and processing conditions.

**Major Comments**

**Comment 1:** How do the authors justify the interpretation of “optimal densification” without using relative density, linear shrinkage, and/or open/closed porosity?

**Response:** The authors aim was to predict the density of composites in the desired processing conditions, since optimization of density would be affected by many other variables (relative density, shrinkage, porosity). The densification was evaluated solely based on the experimentally measured density values of the Cu–Gr composites. The term “optimal densification” has been removed from the manuscript. The processing condition that resulted in the highest measured density among the investigated samples was determined in the present work.

**Comment 2:** How do the authors intend to quantitatively separate the effect of composition from the actual effect of sintering? Without such separation, in what sense can the reported “optimum” be regarded as a processing optimum rather than simply a reflection of the rule of mixtures?

**Response-** We acknowledge that the measured density is inherently influenced by the composite composition, and changes in density may partly arise from the rule of mixtures associated with varying graphite content in addition to the effects of sintering. The present work did not aim to quantitatively decouple the contribution of composition from the intrinsic sintering behavior through parameters such as relative density, densification parameter, shrinkage, or porosity measurements.

Therefore, the term "optimum" refers to the optimum combination of composition and processing parameters within the selected design space rather than a pure sintering optimum. To avoid any ambiguity, the manuscript has been revised to clarify this point and to state that the optimized conditions correspond to the ‘maximum’ predicted density and hardness achieved by RSM within the investigated range of composition and sintering parameters.

**Comment 3:** Is there any quantitative analysis supporting the interpretation of diffusion, neck growth, and the competition between densification and grain growth?

**Response-** We thank the reviewer for this valuable comment. The present study did not include direct quantitative characterization of these phenomena through measurements such as neck-size evolution, grain-size analysis, diffusion coefficients. The experimentally observed trends in density and hardness were discussed according to the well-established principles of powder metallurgy and solid-state sintering reported in the literature.

**Comment 4:** How do the authors explain that, in Table 4, temperature and time have p = 0.0035, while the text states p < 0.0001 for all factors? How do they justify that, in Table 6, term C has p = 0.5170 and is nevertheless treated textually as significant? Which set of R²/adjusted R² values for density should be considered correct: 99.18/98.44% or 99.90/99.40%?

**Response-** The authors thank the reviewer for highlighting the error. The authors have corrected the mistakes in the manuscript and highlighted the same. The correct set of R²/adjusted R² values for density is 99.90/99.40%.

**Comment 5:** What is the quantitative basis for claiming homogeneous graphite dispersion? Were area fraction, interparticle spacing, degree of agglomeration, pore size distribution, or statistical analysis over multiple fields calculated?

**Response-** The authors thank the reviewer for highlighting this important aspect. The assessment of graphite distribution in the present study was based solely on EDX elemental mapping, which provided ‘qualitative’ evidence of the presence and spatial distribution of carbon throughout the examined region of the composite. No quantitative image analysis, such as area fraction measurements, interparticle spacing determination, agglomeration index evaluation, pore size distribution analysis, or statistical assessment over multiple fields of view, was performed.

**Comment 6:** How do the authors demonstrate grain growth in the absence of a quantitative average grain size analysis?

**Response:** We acknowledge that no quantitative grain size analysis was performed in the present study. The authors have removed the discussion regarding evidence of grain growth from the manuscript.

**Comment 7:** How do they infer reduced wettability without contact-angle measurements, more detailed interfacial analysis, or quantitative evidence of interfacial gaps

**Response:** The manuscript has been revised to avoid definitive statements regarding reduced wettability and to present it only as a potential mechanism reported in previous studies. We acknowledge that a rigorous evaluation of wettability would require contact-angle measurements and detailed interfacial analyses, which are beyond the scope of the present investigation. These aspects will be considered in future studies to provide a more comprehensive understanding of the Cu–graphite interfacial behaviour.

**Comment 8:** What is the actual functional relevance of the study for electrical and tribological applications if only density and hardness were measured?

**Response:** The authors thank the reviewer for highlighting this important aspect. However, the current study is focussed on the density and hardness only, the tribological performance of the composites can also be analysed in future work.

**Comment 9:** In what strict technical sense does this work demonstrate sustainable manufacturing? How do the authors justify such wording in the title without any objective sustainability metric? Would it not be more appropriate to replace this formulation with something more modest and methodologically consistent, limited to statistical process optimization?

**Response:** We thank the reviewer for this valuable suggestion. The term “sustainable” has been removed from the title and throughout the manuscript where appropriate. The revised title and text now focus on the actual scope of the study, namely the statistical prediction of properties of the copper-graphite composite using RSM.
