# Supplementary material for: Prediction of the properties of sintered copper-graphite composites using response surface methodology
Source: PLoS One. 2026 Sep 11;21(9):e0357759. doi: 10.1371/journal.pone.0357759 (PMC13567752; doi:10.1371/journal.pone.0357759)
Supplement: Supplementary file 3 [file pone.0357759.s003.docx]

**Response to Reviewer’s comments**

The authors sincerely thank the Editor and the Reviewer for the thorough evaluation of our manuscript and for the constructive comments and valuable suggestions. We greatly appreciate the time and effort devoted to reviewing our work. The comments have been carefully considered, and the manuscript has been comprehensively revised to address all the concerns raised. In particular, we have further strengthened the statistical analysis by clarifying the experimental methodology, improving the discussion on model robustness and limitations, revising the interpretation of the statistical results, and enhancing the transparency of the predictive modelling approach. We believe that these revisions have significantly improved the scientific rigour, clarity, and overall quality of the manuscript. A detailed, point-by-point response to each comment is provided below, and all corresponding changes have been incorporated into the revised manuscript and highlighted for the reviewer's convenience.

**Reviewer #3:**

**Comment 1. Critical Overview:** The manuscript addresses the prediction of sintered density and Vickers microhardness for copper-graphite composites processed via powder metallurgy (PM), employing a central composite design (CCD) coupled with Response Surface Methodology (RSM). The topic holds significant industrial relevance for friction components and electrical contacts. However, the manuscript exhibits flaws, such as the omission of experimental replicates for physical properties and a suspicious level of overfitting in the statistical models that requires thorough justification.

**Response:** The authors thank the reviewer for the careful evaluation of the manuscript and for recognising the industrial relevance of the present work. We appreciate the opportunity to further clarify these important methodological aspects.

Regarding the experimental replicates, we acknowledge that the manuscript did not explicitly describe the replication strategy in sufficient detail. In the present work, each experimental condition defined by the Central Composite Design (CCD) was fabricated once, and the measured physical properties were obtained from multiple measurements on each specimen. Specifically, the Vickers microhardness values reported for each experimental run represent the average of multiple indentations performed at different locations on the specimen surface in order to minimise the influence of local microstructural heterogeneity. Similarly, density was determined using Archimedes' principle following standard practice. We agree that fabrication-level replicates would provide a more rigorous assessment of experimental variability and statistical confidence. However, owing to limitations in material availability and experimental resources, independent replicate specimens for every CCD condition could not be produced. This limitation has now been explicitly acknowledged in the revised manuscript, and it has been stated that future work will include replicated experimental runs to further strengthen the statistical reliability of the developed models.

Regarding the concern of potential overfitting, we fully understand the reviewer's observation that the reported coefficients of determination (R² ≈ 0.99) may appear unusually high. These values primarily arise because the investigated design space was intentionally narrow and carefully controlled, involving only three process variables over limited practical ranges using a Central Composite Design specifically intended for second-order response modelling. To ensure that the high R² values do not merely reflect model overfitting, the revised manuscript now provides a more comprehensive statistical assessment, including Adjusted R², Predicted R², ANOVA, Lack-of-Fit analysis, residual diagnostics, normal probability plots, residual-versus-predicted plots, residual-versus-run-order plots, and predicted-versus-actual plots. These diagnostic analyses collectively indicate that the regression models adequately represent the experimental data within the investigated design space, with randomly distributed residuals, no systematic trends, and statistically insignificant lack-of-fit.

Furthermore, the predictive capability of the developed models was verified through independent confirmation experiments conducted under the optimised processing conditions, in which the experimentally measured density and hardness closely matched the model predictions, with very small prediction errors. We agree that independent external validation or cross-validation with additional experimental datasets would provide a stronger assessment of the model's generalizability. However, such validation was beyond the scope of the present investigation and has now been explicitly acknowledged as a limitation of the study.

Accordingly, the manuscript has been revised to clarify that the developed RSM models are intended to provide reliable prediction and optimisation within the investigated experimental domain, rather than to serve as universal predictive models applicable outside the selected processing window. We believe these revisions substantially strengthen the transparency, statistical rigour, and interpretation of the proposed models while clearly defining their scope and limitations.

**Comment 2. Strengths**

* The use of complementary microstructural characterization techniques (XRD, SEM, and EDS elemental mapping) qualitatively validates the distribution of graphite within the copper matrix.

* The attempt to correlate actual physical parameters (porosity and the difference from theoretical density based on the rule of mixtures) with statistical responses demonstrates a sound physical basis for the discussion of graphite behaviour.

**Response:** The authors are very grateful and obliged for the encouraging comments from the honourable reviewer.

**Comment** **3. Weaknesses and Mandatory Improvements (Major Revisions)**

**3.1 Omission of Replicates (Density)**: While Section 2.2 categorically states that hardness was measured 7 times per sample, the text is completely silent regarding the number of density measurements. Was only one measurement performed per run? The authors must clarify the statistical sample size (N) for density and include error bars in the plots shown in Figures 5, 6, and 7.

**Response-** The authors are thankful for the reviewer’s comment. The sintered density of all composites was measured five times. Also, the density range for each composite is shown as error bars in the respective Figures (5, 6, and 7). The clarification sentence is included in the manuscript and is highlighted.

**Comment** 4. Overfitting Analysis: The R^2^ values presented in Tables 5 and 7 are extremely high (99.9%). However, the predicted and adjusted R^2^ values in the statistical summary tables show unusual variations (e.g., in Table 5, the linear model has an Adj. R^2^ of -0.5364$, whereas the quadratic model jumps to 0.9940). The authors must provide residual diagnostic plots and explain in detail whether the narrow experimental range resulted in an artificially overfitted model.

**Response-** The authors acknowledge the reviewer's comment for highlighting concern regarding potential overfitting and the unusually high R^2^. The apparent variation between the adjusted and predicted R^2^ values across the different models, particularly the low adjusted R2 of the linear model and the substantially higher value for the quadratic model, reflects the ability of the respective model structures to capture the nonlinear response within the investigated experimental domain.

The linear model does not adequately capture the variation in the response, whereas the inclusion of quadratic terms substantially improves the model fit. We acknowledge that the relatively narrow experimental ranges of the input parameters may contribute to the exceptionally high R^2^ values.

We have carefully re-examined the regression models and included the corresponding residual diagnostic plots (9(a, b) and 11(a, b)) to assess model adequacy and potential overfitting. The residual plots show that the residuals are randomly scattered around the zero line without any clear systematic pattern, indicating that the assumptions of model adequacy are reasonably satisfied. Furthermore, the residuals are predominantly distributed within a narrow range, with no evident trend or pronounced outliers. The detailed discussion of residual plots has been added to the manuscript and highlighted.

**Comment 5:** Minor Revisions

* Units of Measurement: In Table 2, the unit for Sintering Temperature is written simply as "C". Correct it to "°C" using the appropriate formatting.

* Scientific Language: In the abstract and throughout the text, eliminate vague and informal terms such as "perfect fit" or "minimal errors". Replace them with precise statistical terms (e.g., "The quadratic model exhibited a strong correlation with experimental data, as evidenced by an R2 of 0.999").

* Time Standardization: Replace the informal abbreviation "hrs" with "h" (International System standard) throughout the text and tables.

**Response:** The authors acknowledge the reviewer comments.

***** Units of Measurement: The unit of sintering temperature in Table 2 is changed to "°C" and highlighted in the manuscript.

* Scientific Language: The authors have replaced the informal terms throughout the manuscript and highlighted the same.

* Time Standardisation: The authors thank the reviewer for pointing this out. The abbreviation “hrs” has been replaced with “h” throughout the manuscript in accordance with the International System of Units.
