# Supplementary material for: Prediction of the properties of sintered copper-graphite composites using response surface methodology
Source: PLoS One. 2026 Sep 11;21(9):e0357759. doi: 10.1371/journal.pone.0357759 (PMC13567752; doi:10.1371/journal.pone.0357759)
Supplement: Supplementary file 4 [file pone.0357759.s004.docx]

**Response to Editor/Reviewers’ comments**

**Comment** **1.** Table 1 should be reworked and error ranges included. At least the equipment error should be considered.

**Response:** We thank the editor for this important suggestion. Table 1 has been revised accordingly, and the corresponding error ranges have been included. The equipment-related measurement errors have been considered and incorporated in the revised table.

**Comment** **2.** JCPDS file numbers corresponding with XRD patterns should be included.

**Response:** The authors are thankful for the valuable suggestion. The corresponding JCPDS reference card numbers have been added to the revised manuscript for the phases identified from the XRD patterns and relevant reference are also included and highlighted in the manuscript.

**Comment** **3.** Fig. 2 has no indicating its repeatability or reproducibility. Besides, is a bulk sample utilized or a powder sample is used?

**Response:** We thank the editor for this thoughtful assessment. Five samples were tested for XRD for better repeatability of results. Also, a bulk sintered samples were utilized for the testing. Also, details are now included in manuscript and highlighted the same.

**Comment** **4.** Discussion of Figs. 3 and 4, at least a new sentence should be included in order to clarify its reproducibility.

**Response:** The authors acknowledge the reviewer comment. The authors have added the sentences in the manuscript and highlighted. To ensure the reproducibility of the microstructural observations, optical and SEM images were obtained from three independently prepared samples under identical experimental and imaging conditions, and the observed microstructural features were found to be consistent across the samples. The details are now included in the manuscript and highlighted the same.

**Comment 5**. The revised text should be verified in order to prevent both US and British English styles. It is verified words in two styles.

**Response:** The revised manuscript has been thoroughly checked to ensure consistent use of American English, and all instances of mixed American and British spelling have been corrected accordingly.

**Thanking you sir!**
