# Supplementary material for: Prediction of the properties of sintered copper-graphite composites using response surface methodology
Source: PLoS One. 2026 Sep 11;21(9):e0357759. doi: 10.1371/journal.pone.0357759 (PMC13567752; doi:10.1371/journal.pone.0357759)
Supplement: Peer review history

# Author comment: Author response to Decision Letter 0

**DOI:** 10.1371/journal.pone.0357759.r001

5 May 2026

Attachment Submitted filename: Response to Reviewers Comments.docx

# Peer review: Decision Letter 0

**DOI:** 10.1371/journal.pone.0357759.r002

**Authors:** Wislei Riuper Osório

28 May 2026

Dear Dr. Katiyar,

Thank you for submitting your manuscript to PLOS ONE. After careful consideration, we feel that it has merit but does not fully meet PLOS ONE’s publication criteria as it currently stands. Therefore, we invite you to submit a revised version of the manuscript that addresses the points raised during the review process.

==============================

**ACADEMIC EDITOR:** Dear Authors;

Please submit your revised manuscript by Jul 27 2026 11:59PM. If you will need more time than this to complete your revisions, please reply to this message or contact the journal office at plosone@plos.org. When you're ready to submit your revision, log on to https://www.editorialmanager.com/pone/ and select the 'Submissions Needing Revision' folder to locate your manuscript file.

- A letter that responds to each point raised by the academic editor and reviewer(s). You should upload this letter as a separate file labeled 'Response to Reviewers'.
- A marked-up copy of your manuscript that highlights changes made to the original version. You should upload this as a separate file labeled 'Revised Manuscript with Track Changes'.
- An unmarked version of your revised paper without tracked changes. You should upload this as a separate file labeled 'Manuscript'.

If you would like to make changes to your financial disclosure, please include your updated statement in your cover letter. Guidelines for resubmitting your figure files are available below the reviewer comments at the end of this letter.

If applicable, we recommend that you deposit your laboratory protocols in protocols.io to enhance the reproducibility of your results. Protocols.io assigns your protocol its own identifier (DOI) so that it can be cited independently in the future. For instructions see: https://journals.plos.org/plosone/s/submission-guidelines#loc-laboratory-protocols. Additionally, PLOS ONE offers an option for publishing peer-reviewed Lab Protocol articles, which describe protocols hosted on protocols.io. Read more information on sharing protocols at https://plos.org/protocols?utm_medium=editorial-email&utm_source=authorletters&utm_campaign=protocols.

As the corresponding author, your ORCID iD is verified in the submission system and will appear in the published article. PLOS supports the use of ORCID, and we encourage all coauthors to register for an ORCID iD and use it as well. Please encourage your coauthors to verify their ORCID iD within the submission system before final acceptance, as unverified ORCID iDs will not appear in the published article. *Only*  the individual author can complete the verification step; PLOS staff *cannot*  verify ORCID iDs on behalf of authors.

We look forward to receiving your revised manuscript.

Kind regards,

Wislei Riuper Osório

Academic Editor

PLOS One

Journal Requirements:

When submitting your revision, we need you to address these additional requirements.

1. Please ensure that your manuscript meets PLOS ONE's style requirements, including those for file naming. The PLOS ONE style templates can be found at https://journals.plos.org/plosone/s/file?id=wjVg/PLOSOne_formatting_sample_main_body.pdf and https://journals.plos.org/plosone/s/file?id=ba62/PLOSOne_formatting_sample_title_authors_affiliations.pdf

2. Please note that PLOS One has specific guidelines on code sharing for submissions in which author-generated code underpins the findings in the manuscript. In these cases, we expect all author-generated code to be made available without restrictions upon publication of the work. Please review our guidelines at https://journals.plos.org/plosone/s/materials-and-software-sharing#loc-sharing-code and ensure that your code is shared in a way that follows best practice and facilitates reproducibility and reuse.

3. We note that Figure 1 in your submission contains images which may be copyrighted. All PLOS content is published under the Creative Commons Attribution License (CC BY 4.0), which means that the manuscript, images, and Supporting Information files will be freely available online, and any third party is permitted to access, download, copy, distribute, and use these materials in any way, even commercially, with proper attribution. For more information, see our copyright guidelines: http://journals.plos.org/plosone/s/licenses-and-copyright.

We require you to either (1) present written permission from the copyright holder to publish these figures specifically under the CC BY 4.0 license, or (2) remove the figures from your submission:

1. You may seek permission from the original copyright holder of Figure 1 to publish the content specifically under the CC BY 4.0 license.

We recommend that you contact the original copyright holder with the Content Permission Form (http://journals.plos.org/plosone/s/file?id=7c09/content-permission-form.pdf) and the following text:

“I request permission for the open-access journal PLOS ONE to publish XXX under the Creative Commons Attribution License (CCAL) CC BY 4.0 (http://creativecommons.org/licenses/by/4.0/). Please be aware that this license allows unrestricted use and distribution, even commercially, by third parties. Please reply and provide explicit written permission to publish XXX under a CC BY license and complete the attached form.”

Please upload the completed Content Permission Form or other proof of granted permissions as an "Other" file with your submission.

In the figure caption of the copyrighted figure, please include the following text: “Reprinted from [ref] under a CC BY license, with permission from [name of publisher], original copyright [original copyright year].”

2. If you are unable to obtain permission from the original copyright holder to publish these figures under the CC BY 4.0 license or if the copyright holder’s requirements are incompatible with the CC BY 4.0 license, please either i) remove the figure or ii) supply a replacement figure that complies with the CC BY 4.0 license. Please check copyright information on all replacement figures and update the figure caption with source information. If applicable, please specify in the figure caption text when a figure is similar but not identical to the original image and is therefore for illustrative purposes only.

If the reviewer comments include a recommendation to cite specific previously published works, please review and evaluate these publications to determine whether they are relevant and should be cited. There is no requirement to cite these works unless the editor has indicated otherwise.

Additional Editor Comments:

Dear Authors;

Based on the Reviewers’ comments and suggestions, the proposed manuscript should meticulously be revised and improved. For this, it is indicated that a Rebuttal letter be prepared. Please, taken attention in each one of the items comments. Additionally, it is hardly suggested that a Grammar and Spelling revision be also made. Besides, reproducibility and repeatability should also be better explained and elucidated.

[Note: HTML markup is below. Please do not edit.]

Reviewers' comments:

Reviewer's Responses to Questions

**Comments to the Author**

1. Is the manuscript technically sound, and do the data support the conclusions?

Reviewer #1: Partly

Reviewer #2: Partly

**********

2. Has the statistical analysis been performed appropriately and rigorously?

Reviewer #1: No

Reviewer #2: No

**********

3. Have the authors made all data underlying the findings in their manuscript fully available?

The PLOS Data policy

Reviewer #1: No

Reviewer #2: Yes

**********

4. Is the manuscript presented in an intelligible fashion and written in standard English?

Reviewer #1: No

Reviewer #2: Yes

**********

Reviewer #1: I previously reviewed an earlier version of this manuscript, and although the revised submission shows noticeable improvements in organization, statistical presentation, and discussion of some physical mechanisms, several important concerns raised in the previous review remain insufficiently addressed. In my opinion, these unresolved issues are substantial and still justify a recommendation of major revision.

Important methodological and reproducibility concerns remain unresolved.

a. Robustness and predictive reliability of the statistical model

The manuscript continues to report extremely high R² values (~0.999) obtained from a relatively limited experimental design space. Although the revised version now briefly acknowledges the possibility of overfitting, no independent external validation or cross-validation procedure has been performed. The validation remains restricted to confirmation experiments conducted very close to the optimized condition itself, which is not sufficient to fully demonstrate the true predictive capability or generalizability of the proposed model. Since the work strongly emphasizes predictive modelling and optimization, stronger validation procedures are necessary to support these claims.

b) Experimental reproducibility and data availability

The manuscript still states only that “Data is provided within the manuscript. ” However, no structured raw dataset, supplementary spreadsheet, or publicly accessible repository has been provided. Considering that the study relies heavily on ANOVA/RSM modelling and statistical optimization, the absence of openly accessible experimental datasets substantially limits reproducibility, transparency, and independent verification of the reported results. This remains an important concern, particularly under the reproducibility and data-sharing standards expected by PLOS ONE.

C. Scientific contribution and scope

While the manuscript has improved its discussion and presentation, the core contribution still remains largely centered on the application of established statistical optimization techniques (RSM/ANOVA) to a relatively restricted processing window of Cu–Gr composites. The broader scientific impact and methodological novelty of the study could still be more clearly established.

D. Language and scientific writing

Although improved compared with the previous version, the manuscript still contains several grammatical inconsistencies and awkward sentence constructions throughout the text. Additional professional language revision would improve clarity and readability.

the remaining concerns regarding validation robustness, predictive reliability, reproducibility, and data transparency are still significant and should be more thoroughly addressed before the manuscript can be considered for publication.

Reviewer #2: The manuscript addresses an applied and industrially relevant topic and is currently in its second revision; however, in its present form, it still falls short of the scientific rigor required to support its central claims of “predictive modelling,” “optimization,” and “sustainable manufacturing.” The revision carried out by the authors has improved certain aspects of writing and transparency, but it has not resolved the core conceptual and methodological problems previously identified by the reviewers. The study remains essentially based on a quadratic RSM/CCD fit over a restricted experimental space, with a predominantly qualitative physical interpretation and internal statistical inconsistencies that are still present in the revised text. For example, one problematic issue remains the choice of absolute density as a response variable while the graphite content is simultaneously varied. The manuscript itself acknowledges that density decreases with increasing graphite content due to the intrinsic difference between the densities of copper and graphite, in addition to possible effects of wettability and agglomeration. This means that the “density” response is inevitably confounded by the rule of mixtures, without adequately isolating the actual densification efficiency. Under these conditions, the interpretation of the processing optimum becomes conceptually weakened. Furthermore, although the authors claim to have corrected the ANOVA and strengthened the statistical validation, the revised text still contains internal inconsistencies. In the density ANOVA, temperature and time appear with p = 0.0035, yet the text states that all three factors have p < 0.0001; in the hardness ANOVA, term C (holding time) appears with p = 0.5170, and is therefore not significant, but the text states that A, B, and C are significant with p < 0.0001. There is also inconsistency in the R² and adjusted R² values discussed for the density model. This compromises the reliability of the statistical analysis.

In addition:

1. How do the authors justify the interpretation of “optimal densification” without using relative density, linear shrinkage, and/or open/closed porosity?

2. How do the authors intend to quantitatively separate the effect of composition from the actual effect of sintering? Without such separation, in what sense can the reported “optimum” be regarded as a processing optimum rather than simply a reflection of the rule of mixtures?

3. Is there any quantitative analysis supporting the interpretation of diffusion, neck growth, and the competition between densification and grain growth?

4. How do the authors explain that, in Table 4, temperature and time have p = 0.0035, while the text states p < 0.0001 for all factors? How do they justify that, in Table 6, term C has p = 0.5170 and is nevertheless treated textually as significant? Which set of R²/adjusted R² values for density should be considered correct: 99.18/98.44% or 99.90/99.40%?

5. What is the quantitative basis for claiming homogeneous graphite dispersion? Were area fraction, interparticle spacing, degree of agglomeration, pore size distribution, or statistical analysis over multiple fields calculated?

6. How do the authors demonstrate grain growth in the absence of a quantitative average grain size analysis?

7. How do they infer reduced wettability without contact-angle measurements, more detailed interfacial analysis, or quantitative evidence of interfacial gaps?

8. What is the actual functional relevance of the study for electrical and tribological applications if only density and hardness were measured?

9. In what strict technical sense does this work demonstrate sustainable manufacturing? How do the authors justify such wording in the title without any objective sustainability metric? Would it not be more appropriate to replace this formulation with something more modest and methodologically consistent, limited to statistical process optimization?

**********

what does this mean?). If published, this will include your full peer review and any attached files.

If you choose “no”, your identity will remain anonymous but your review may still be made public.

**Do you want your identity to be public for this peer review?**  For information about this choice, including consent withdrawal, please see our Privacy Policy

Reviewer #1: No

Reviewer #2: No

**********

[NOTE: If reviewer comments were submitted as an attachment file, they will be attached to this email and accessible via the submission site. Please log into your account, locate the manuscript record, and check for the action link "View Attachments". If this link does not appear, there are no attachment files.]

To ensure your figures meet our technical requirements, please review our figure guidelines: https://journals.plos.org/plosone/s/figures

You may also use PLOS’s free figure tool, NAAS, to help you prepare publication quality figures: https://journals.plos.org/plosone/s/figures#loc-tools-for-figure-preparation.

NAAS will assess whether your figures meet our technical requirements by comparing each figure against our figure specifications.

# Author comment: Author response to Decision Letter 1

**DOI:** 10.1371/journal.pone.0357759.r003

30 Jun 2026

Journal Requirements

When submitting your revision, we need you to address these additional requirements.

1. Please ensure that your manuscript meets PLOS ONE's style requirements, including those for file naming. The PLOS ONE style templates can be found at https://journals.plos.org/plosone/s/file?id=wjVg/PLOSOne_formatting_sample_main_body.pdf and https://journals.plos.org/plosone/s/file?id=ba62/PLOSOne_formatting_sample_title_authors_affiliations.pdf

Response: The manuscript has been structured according to PLOS ONE's style requirements.

2. Please note that PLOS One has specific guidelines on code sharing for submissions in which author-generated code underpins the findings in the manuscript. In these cases, we expect all author-generated code to be made available without restrictions upon publication of the work. Please review our guidelines at https://journals.plos.org/plosone/s/materials-and-software-sharing#loc-sharing-code and ensure that your code is shared in a way that follows best practice and facilitates reproducibility and reuse.

Response: The authors have complied according to the guidelines.

3. We note that Figure 1 in your submission contains images which may be copyrighted. All PLOS content is published under the Creative Commons Attribution License (CC BY 4.0), which means that the manuscript, images, and Supporting Information files will be freely available online, and any third party is permitted to access, download, copy, distribute, and use these materials in any way, even commercially, with proper attribution. For more information, see our copyright guidelines: http://journals.plos.org/plosone/s/licenses-and-copyright.

We require you to either (1) present written permission from the copyright holder to publish these figures specifically under the CC BY 4.0 license, or (2) remove the figures from your submission:

Response: Figure 1 of the manuscript contain images that are originally taken by the author. The authors have worked on the ball milling machine and furnace shown in the Figure 1.

General Editorial Questions

Comment 1: Is the manuscript technically sound, and do the data support the conclusions?

Reviewer #1: Partly

Reviewer #2: Partly

Response: The manuscript has been revised to improve technical clarity and strengthen the linkage between experimental data and conclusions. Additional explanations have been incorporated to better justify the observed trends, and limitations of the study have now been explicitly acknowledged. While the experimental framework remains valid, the discussion has been expanded to avoid overgeneralization and to ensure that conclusions are fully supported by the presented data.

Comment 2: Has the statistical analysis been performed appropriately and rigorously?

Reviewer #1: No

Reviewer #2: No

Response: We appreciate the reviewers’ observations. The statistical analysis has been carefully re-evaluated. Errors and inconsistencies in the ANOVA tables have been corrected, and additional statistical validation—such as residual analysis and diagnostic plots—has now been included. The interpretation of statistical parameters has also been revised to align with standard practices.

Comment 3: Have the authors made all data underlying the findings fully available?

Reviewer #1: No

Reviewer #2: Yes

Response: All relevant data supporting the findings are included within the manuscript. The experimental data of density and hardness are shown in Figures 4-6. In addition, structured datasets and supporting numerical values have now been organized more clearly (given in Table 3).

Comment 4: Is the manuscript written in standard English and presented clearly?

Reviewer #1: No

Reviewer #2: Yes

Response: The manuscript has undergone thorough language editing to improve clarity, grammar, and readability. Redundant statements have been removed, and the overall structure has been refined to ensure clear and concise scientific communication.

Reviewer #1:

I previously reviewed an earlier version of this manuscript, and although the revised submission shows noticeable improvements in organization, statistical presentation, and discussion of some physical mechanisms, several important concerns raised in the previous review remain insufficiently addressed. In my opinion, these unresolved issues are substantial and still justify a recommendation of major revision.

Important methodological and reproducibility concerns remain unresolved.

Major´s

Comment 1. Robustness and predictive reliability of the statistical model

The manuscript continues to report extremely high R² values (~0.999) obtained from a relatively limited experimental design space. Although the revised version now briefly acknowledges the possibility of overfitting, no independent external validation or cross-validation procedure has been performed. The validation remains restricted to confirmation experiments conducted very close to the optimized condition itself, which is not sufficient to fully demonstrate the true predictive capability or generalizability of the proposed model. Since the work strongly emphasizes predictive modelling and optimization, stronger validation procedures are necessary to support these claims

Response: We thank the reviewer for this important comment. In the present study, the models were developed using Response Surface Methodology (RSM) based on the experimental data generated within the investigated design space, and their adequacy was evaluated through analysis of variance (ANOVA), residual analysis and predicted-versus-actual plots. However, additional validation approaches, such as cross-validation, independent test datasets, or experiments conducted at new design points outside the optimization region, would strengthen confidence in the model's generalizability. These aspects are beyond the scope of the present study and will be considered in future work

Comment 2. Experimental reproducibility and data availability

The manuscript still states only that “Data is provided within the manuscript. ” However, no structured raw dataset, supplementary spreadsheet, or publicly accessible repository has been provided. Considering that the study relies heavily on ANOVA/RSM modelling and statistical optimization, the absence of openly accessible experimental datasets substantially limits reproducibility, transparency, and independent verification of the reported results. This remains an important concern, particularly under the reproducibility and data-sharing standards expected by PLOS ONE.

Response: The experimental data is provided in the Table 3 for both density and hardness of the composite.

Comment 3. Scientific contribution and scope

While the manuscript has improved its discussion and presentation, the core contribution still remains largely centered on the application of established statistical optimization techniques (RSM/ANOVA) to a relatively restricted processing window of Cu–Gr composites. The broader scientific impact and methodological novelty of the study could still be more clearly established.

Response- We thank the reviewer for this thoughtful assessment and constructive suggestion. The primary objective of the present study was not to develop a new statistical methodology but rather to systematically investigate the combined effects of graphite content, sintering temperature, and sintering time on the density and hardness of Cu–Gr composites and to establish empirical relationships between these processing variables and the resulting properties. The contribution of the work lies in providing a statistically guided optimization framework for Cu–Gr composites fabricated through powder metallurgy and in identifying the parameter combinations that maximize the measured responses within the investigated experimental domain. Further, the RSM technique can be used for other metal matrix composites, also for analysis of tribological performance the composites.

Comment 4. Language and scientific writing

Although improved compared with the previous version, the manuscript still contains several grammatical inconsistencies and awkward sentence constructions throughout the text. Additional professional language revision would improve clarity and readability.

the remaining concerns regarding validation robustness, predictive reliability, reproducibility, and data transparency are still significant and should be more thoroughly addressed before the manuscript can be considered for publication.

Response- The authors acknowledge the reviewer comment. The manuscript has undergone a thorough revision to improve grammar, sentence structure, clarity, and overall readability. The text has been carefully reviewed, and several grammatical inconsistencies have been corrected throughout the manuscript. With respect to the concerns related to validation robustness, predictive reliability, reproducibility, and data transparency, we acknowledge the importance of these aspects. The scope and limitations of the developed RSM models have been further clarified in the revised manuscript.

Reviewer #2:

The manuscript addresses an applied and industrially relevant topic and is currently in its second revision; however, in its present form, it still falls short of the scientific rigor required to support its central claims of “predictive modelling,” “optimization,” and “sustainable manufacturing.” The revision carried out by the authors has improved certain aspects of writing and transparency, but it has not resolved the core conceptual and methodological problems previously identified by the reviewers. The study remains essentially based on a quadratic RSM/CCD fit over a restricted experimental space, with a predominantly qualitative physical interpretation and internal statistical inconsistencies that are still present in the revised text. For example, one problematic issue remains the choice of absolute density as a response variable while the graphite content is simultaneously varied. The manuscript itself acknowledges that density decreases with increasing graphite content due to the intrinsic difference between the densities of copper and graphite, in addition to possible effects of wettability and agglomeration. This means that the “density” response is inevitably confounded by the rule of mixtures, without adequately isolating the actual densification efficiency. Under these conditions, the interpretation of the processing optimum becomes conceptually weakened. Furthermore, although the authors claim to have corrected the ANOVA and strengthened the statistical validation, the revised text still contains internal inconsistencies. In the density ANOVA, temperature and time appear with p = 0.0035, yet the text states that all three factors have p < 0.0001; in the hardness ANOVA, term C (holding time) appears with p = 0.5170, and is therefore not significant, but the text states that A, B, and C are significant with p < 0.0001. There is also inconsistency in the R² and adjusted R² values discussed for the density model. This compromises the reliability of the statistical analysis.

Response: The authors sincerely thank the reviewer for the careful and constructive assessment of the manuscript. We acknowledge that several statements in the previous version generalized the significance of model terms and inadvertently introduced inconsistencies between the ANOVA tables and their interpretation. The manuscript has been thoroughly revised to ensure complete consistency between the statistical outputs and the accompanying discussion.

For the density model, the previous statement indicating that all three factors possessed p-values lower than 0.0001 has been corrected. The revised text now accurately reports that composition (A), temperature (B), and holding time (C) are statistically significant with p-values of <0.0001, 0.0035, and 0.0035, respectively. Likewise, the values of R² and adjusted R² have been carefully verified and corrected wherever necessary. Similarly, for the hardness model, the previous interpretation has been revised to reflect the actual ANOVA results. The linear effect of holding time (C) was found to be statistically insignificant (p = 0.5170), and therefore the earlier statement suggesting that all three linear factors possessed p-values lower than 0.0001 has been removed. The revised discussion now clarifies that the effect of holding time on hardness is predominantly nonlinear, as evidenced by the highly significant quadratic term (C², p < 0.0001) and the significant BC interaction term (p = 0.0002). Consequently, holding time contributes to the hardness response through curvature and interaction effects rather than through a simple linear relationship.

In addition, the sentence referring to possible inconsistencies arising from rounding of numerical values has been removed to avoid ambiguity and to maintain the integrity of the statistical analysis. With regard to the reviewer's concern regarding the use of density as a response variable, the authors agree that the absolute density of Cu–graphite composites is inherently influenced by the density difference between copper and graphite according to the rule of mixtures. Therefore, a reduction in density with increasing graphite content cannot be interpreted solely as a reduction in densification efficiency. To address this concern, the discussion has been revised to emphasize that the measured density values represent the combined influence of composition and processing conditions.

Major Comments

Comment 1: How do the authors justify the interpretation of “optimal densification” without using relative density, linear shrinkage, and/or open/closed porosity?

Response: The authors aim was to predict the density of composites in the desired processing conditions, since optimization of density would be affected by many other variables (relative density, shrinkage, porosity). The densification was evaluated solely based on the experimentally measured density values of the Cu–Gr composites. The term “optimal densification” has been removed from the manuscript. The processing condition that resulted in the highest measured density among the investigated samples was determined in the present work.

Comment 2: How do the authors intend to quantitatively separate the effect of composition from the actual effect of sintering? Without such separation, in what sense can the reported “optimum” be regarded as a processing optimum rather than simply a reflection of the rule of mixtures?

Response- We acknowledge that the measured density is inherently influenced by the composite composition, and changes in density may partly arise from the rule of mixtures associated with varying graphite content in addition to the effects of sintering. The present work did not aim to quantitatively decouple the contribution of composition from the intrinsic sintering behavior through parameters such as relative density, densification parameter, shrinkage, or porosity measurements.

Therefore, the term "optimum" refers to the optimum combination of composition and processing parameters within the selected design space rather than a pure sintering optimum. To avoid any ambiguity, the manuscript has been revised to clarify this point and to state that the optimized conditions correspond to the ‘maximum’ predicted density and hardness achieved by RSM within the investigated range of composition and sintering parameters.

Comment 3: Is there any quantitative analysis supporting the interpretation of diffusion, neck growth, and the competition between densification and grain growth?

Response- We thank the reviewer for this valuable comment. The present study did not include direct quantitative characterization of these phenomena through measurements such as neck-size evolution, grain-size analysis, diffusion coefficients. The experimentally observed trends in density and hardness were discussed according to the well-established principles of powder metallurgy and solid-state sintering reported in the literature.

Comment 4: How do the authors explain that, in Table 4, temperature and time have p = 0.0035, while the text states p < 0.0001 for all factors? How do they justify that, in Table 6, term C has p = 0.5170 and is nevertheless treated textually as si

Attachment Submitted filename: Response to the Reviewers.docx

# Peer review: Decision Letter 1

**DOI:** 10.1371/journal.pone.0357759.r004

**Authors:** Wislei Riuper Osório

22 Jul 2026

Dear Dr. Katiyar,

Thank you for submitting your manuscript to PLOS ONE. After careful consideration, we feel that it has merit but does not fully meet PLOS ONE’s publication criteria as it currently stands. Therefore, we invite you to submit a revised version of the manuscript that addresses the points raised during the review process.

==============================

**ACADEMIC EDITOR:**

Dear Authors;

Please, in this second round of the revision, Reviewers have again provided comments concern to statistical aspects and repeability concerning to data attained. Please, it should meticulusly revised and improved. For this purpose, please, consider each one of the comments provided and a rebutal should be uploaded. Based on these Reviewer's comments, it is expected that Authors provide new sentences explaining details and procedures about the commnets provided. After this, it seems that the manuscript will deserve its publication.

==============================

Please submit your revised manuscript by Sep 20 2026 11:59PM. If you will need more time than this to complete your revisions, please reply to this message or contact the journal office at plosone@plos.org. When you're ready to submit your revision, log on to https://www.editorialmanager.com/pone/ and select the 'Submissions Needing Revision' folder to locate your manuscript file.

- A letter that responds to each point raised by the academic editor and reviewer(s). You should upload this letter as a separate file labeled 'Response to Reviewers'.
- A marked-up copy of your manuscript that highlights changes made to the original version. You should upload this as a separate file labeled 'Revised Manuscript with Track Changes'.
- An unmarked version of your revised paper without tracked changes. You should upload this as a separate file labeled 'Manuscript'.

If you would like to make changes to your financial disclosure, please include your updated statement in your cover letter. Guidelines for resubmitting your figure files are available below the reviewer comments at the end of this letter.

If applicable, we recommend that you deposit your laboratory protocols in protocols.io to enhance the reproducibility of your results. Protocols.io assigns your protocol its own identifier (DOI) so that it can be cited independently in the future. For instructions see: https://journals.plos.org/plosone/s/submission-guidelines#loc-laboratory-protocols. Additionally, PLOS ONE offers an option for publishing peer-reviewed Lab Protocol articles, which describe protocols hosted on protocols.io. Read more information on sharing protocols at https://plos.org/protocols?utm_medium=editorial-email&utm_source=authorletters&utm_campaign=protocols.

As the corresponding author, your ORCID iD is verified in the submission system and will appear in the published article. PLOS supports the use of ORCID, and we encourage all coauthors to register for an ORCID iD and use it as well. Please encourage your coauthors to verify their ORCID iD within the submission system before final acceptance, as unverified ORCID iDs will not appear in the published article. *Only*  the individual author can complete the verification step; PLOS staff *cannot*  verify ORCID iDs on behalf of authors.

We look forward to receiving your revised manuscript.

Kind regards,

Wislei Riuper Osório

Academic Editor

PLOS One

Journal Requirements:

If the reviewer comments include a recommendation to cite specific previously published works, please review and evaluate these publications to determine whether they are relevant and should be cited. There is no requirement to cite these works unless the editor has indicated otherwise.

Additional Editor Comments (if provided):

Dear Authors;

Please, in this second round of the revision, Reviewers have again provided comments concern to statistical aspects and repeability concerning to data attained. Please, it should meticulusly revised and improved. For this purpose, please, consider each one of the comments provided and a rebutal should be uploaded. Based on these Reviewer's comments, it is expected that Authors provide new sentences explaining details and procedures about the comments provided. After this, it seems that the manuscript will deserve its publication.

[Note: HTML markup is below. Please do not edit.]

Reviewers' comments:

Reviewer's Responses to Questions

**Comments to the Author**

Reviewer #3: (No Response)

**********

2. Is the manuscript technically sound, and do the data support the conclusions?

Reviewer #3: Partly

**********

3. Has the statistical analysis been performed appropriately and rigorously?

Reviewer #3: (No Response)

**********

4. Have the authors made all data underlying the findings in their manuscript fully available?

The PLOS Data policy

Reviewer #3: (No Response)

**********

5. Is the manuscript presented in an intelligible fashion and written in standard English?

Reviewer #3: Yes

**********

Reviewer #3: 1. Critical Overview

The manuscript addresses the prediction of sintered density and Vickers microhardness for copper-graphite composites processed via powder metallurgy (PM), employing a central composite design (CCD) coupled with Response Surface Methodology (RSM). The topic holds significant industrial relevance for friction components and electrical contacts. However, the manuscript exhibits flaws, such as the omission of experimental replicates for physical properties and a suspicious level of overfitting in the statistical models that requires thorough justification.

2. Strengths

* The use of complementary microstructural characterization techniques (XRD, SEM, and EDS elemental mapping) qualitatively validates the distribution of graphite within the copper matrix.

* The attempt to correlate actual physical parameters (porosity and the difference from theoretical density based on the rule of mixtures) with statistical responses demonstrates a sound physical basis for the discussion of graphite behavior.

3. Weaknesses and Mandatory Improvements (Major Revisions)

* Omission of Replicates (Density): While Section 2.2 categorically states that hardness was measured 7 times per sample, the text is completely silent regarding the number of density measurements. Was only one measurement performed per run? The authors must clarify the statistical sample size (N) for density and include error bars in the plots shown in Figures 5, 6, and 7.

* Overfitting Analysis: The R2 values presented in Tables 5 and 7 are extremely high (99.9%). However, the predicted and adjusted R2 values in the statistical summary tables show unusual variations (e.g., in Table 5, the linear model has an Adj. R2 of -0.5364$, whereas the quadratic model jumps to 0.9940). The authors must provide residual diagnostic plots and explain in detail whether the narrow experimental range resulted in an artificially overfitted model.

4. Minor Revisions

* Units of Measurement: In Table 2, the unit for Sintering Temperature is written simply as "C". Correct it to "°C" using the appropriate formatting.

* Scientific Language: In the abstract and throughout the text, eliminate vague and informal terms such as "perfect fit" or "minimal errors". Replace them with precise statistical terms (e.g., "The quadratic model exhibited a strong correlation with experimental data, as evidenced by an R2 of 0.999").

* Time Standardization: Replace the informal abbreviation "hrs" with "h" (International System standard) throughout the text and tables.

**********

what does this mean?). If published, this will include your full peer review and any attached files.

If you choose “no”, your identity will remain anonymous but your review may still be made public.

**Do you want your identity to be public for this peer review?**  For information about this choice, including consent withdrawal, please see our Privacy Policy

Reviewer #3: No

**********

[NOTE: If reviewer comments were submitted as an attachment file, they will be attached to this email and accessible via the submission site. Please log into your account, locate the manuscript record, and check for the action link "View Attachments". If this link does not appear, there are no attachment files.]

To ensure your figures meet our technical requirements, please review our figure guidelines: https://journals.plos.org/plosone/s/figures

You may also use PLOS’s free figure tool, NAAS, to help you prepare publication quality figures: https://journals.plos.org/plosone/s/figures#loc-tools-for-figure-preparation.

NAAS will assess whether your figures meet our technical requirements by comparing each figure against our figure specifications.

# Author comment: Author response to Decision Letter 2

**DOI:** 10.1371/journal.pone.0357759.r005

5 Aug 2026

Response to Reviewer’s comments

The authors sincerely thank the Editor and the Reviewer for the thorough evaluation of our manuscript and for the constructive comments and valuable suggestions. We greatly appreciate the time and effort devoted to reviewing our work. The comments have been carefully considered, and the manuscript has been comprehensively revised to address all the concerns raised. In particular, we have further strengthened the statistical analysis by clarifying the experimental methodology, improving the discussion on model robustness and limitations, revising the interpretation of the statistical results, and enhancing the transparency of the predictive modelling approach. We believe that these revisions have significantly improved the scientific rigour, clarity, and overall quality of the manuscript. A detailed, point-by-point response to each comment is provided below, and all corresponding changes have been incorporated into the revised manuscript and highlighted for the reviewer's convenience.

Reviewer #3:

Comment 1. Critical Overview: The manuscript addresses the prediction of sintered density and Vickers microhardness for copper-graphite composites processed via powder metallurgy (PM), employing a central composite design (CCD) coupled with Response Surface Methodology (RSM). The topic holds significant industrial relevance for friction components and electrical contacts. However, the manuscript exhibits flaws, such as the omission of experimental replicates for physical properties and a suspicious level of overfitting in the statistical models that requires thorough justification.

Response: The authors thank the reviewer for the careful evaluation of the manuscript and for recognising the industrial relevance of the present work. We appreciate the opportunity to further clarify these important methodological aspects.

Regarding the experimental replicates, we acknowledge that the manuscript did not explicitly describe the replication strategy in sufficient detail. In the present work, each experimental condition defined by the Central Composite Design (CCD) was fabricated once, and the measured physical properties were obtained from multiple measurements on each specimen. Specifically, the Vickers microhardness values reported for each experimental run represent the average of multiple indentations performed at different locations on the specimen surface in order to minimise the influence of local microstructural heterogeneity. Similarly, density was determined using Archimedes' principle following standard practice. We agree that fabrication-level replicates would provide a more rigorous assessment of experimental variability and statistical confidence. However, owing to limitations in material availability and experimental resources, independent replicate specimens for every CCD condition could not be produced. This limitation has now been explicitly acknowledged in the revised manuscript, and it has been stated that future work will include replicated experimental runs to further strengthen the statistical reliability of the developed models.

Regarding the concern of potential overfitting, we fully understand the reviewer's observation that the reported coefficients of determination (R² ≈ 0.99) may appear unusually high. These values primarily arise because the investigated design space was intentionally narrow and carefully controlled, involving only three process variables over limited practical ranges using a Central Composite Design specifically intended for second-order response modelling. To ensure that the high R² values do not merely reflect model overfitting, the revised manuscript now provides a more comprehensive statistical assessment, including Adjusted R², Predicted R², ANOVA, Lack-of-Fit analysis, residual diagnostics, normal probability plots, residual-versus-predicted plots, residual-versus-run-order plots, and predicted-versus-actual plots. These diagnostic analyses collectively indicate that the regression models adequately represent the experimental data within the investigated design space, with randomly distributed residuals, no systematic trends, and statistically insignificant lack-of-fit.

Furthermore, the predictive capability of the developed models was verified through independent confirmation experiments conducted under the optimised processing conditions, in which the experimentally measured density and hardness closely matched the model predictions, with very small prediction errors. We agree that independent external validation or cross-validation with additional experimental datasets would provide a stronger assessment of the model's generalizability. However, such validation was beyond the scope of the present investigation and has now been explicitly acknowledged as a limitation of the study.

Accordingly, the manuscript has been revised to clarify that the developed RSM models are intended to provide reliable prediction and optimisation within the investigated experimental domain, rather than to serve as universal predictive models applicable outside the selected processing window. We believe these revisions substantially strengthen the transparency, statistical rigour, and interpretation of the proposed models while clearly defining their scope and limitations.

Comment 2. Strengths

* The use of complementary microstructural characterization techniques (XRD, SEM, and EDS elemental mapping) qualitatively validates the distribution of graphite within the copper matrix.

* The attempt to correlate actual physical parameters (porosity and the difference from theoretical density based on the rule of mixtures) with statistical responses demonstrates a sound physical basis for the discussion of graphite behaviour.

Response: The authors are very grateful and obliged for the encouraging comments from the honourable reviewer.

Comment 3. Weaknesses and Mandatory Improvements (Major Revisions)

3.1 Omission of Replicates (Density): While Section 2.2 categorically states that hardness was measured 7 times per sample, the text is completely silent regarding the number of density measurements. Was only one measurement performed per run? The authors must clarify the statistical sample size (N) for density and include error bars in the plots shown in Figures 5, 6, and 7.

Response- The authors are thankful for the reviewer’s comment. The sintered density of all composites was measured five times. Also, the density range for each composite is shown as error bars in the respective Figures (5, 6, and 7). The clarification sentence is included in the manuscript and is highlighted.

Comment 4. Overfitting Analysis: The R2 values presented in Tables 5 and 7 are extremely high (99.9%). However, the predicted and adjusted R2 values in the statistical summary tables show unusual variations (e.g., in Table 5, the linear model has an Adj. R2 of -0.5364$, whereas the quadratic model jumps to 0.9940). The authors must provide residual diagnostic plots and explain in detail whether the narrow experimental range resulted in an artificially overfitted model.

Response- The authors acknowledge the reviewer's comment for highlighting concern regarding potential overfitting and the unusually high R2. The apparent variation between the adjusted and predicted R2 values across the different models, particularly the low adjusted R2 of the linear model and the substantially higher value for the quadratic model, reflects the ability of the respective model structures to capture the nonlinear response within the investigated experimental domain.

The linear model does not adequately capture the variation in the response, whereas the inclusion of quadratic terms substantially improves the model fit. We acknowledge that the relatively narrow experimental ranges of the input parameters may contribute to the exceptionally high R2 values.

We have carefully re-examined the regression models and included the corresponding residual diagnostic plots (9(a, b) and 11(a, b)) to assess model adequacy and potential overfitting. The residual plots show that the residuals are randomly scattered around the zero line without any clear systematic pattern, indicating that the assumptions of model adequacy are reasonably satisfied. Furthermore, the residuals are predominantly distributed within a narrow range, with no evident trend or pronounced outliers. The detailed discussion of residual plots has been added to the manuscript and highlighted.

Comment 5: Minor Revisions

* Units of Measurement: In Table 2, the unit for Sintering Temperature is written simply as "C". Correct it to "°C" using the appropriate formatting.

* Scientific Language: In the abstract and throughout the text, eliminate vague and informal terms such as "perfect fit" or "minimal errors". Replace them with precise statistical terms (e.g., "The quadratic model exhibited a strong correlation with experimental data, as evidenced by an R2 of 0.999").

* Time Standardization: Replace the informal abbreviation "hrs" with "h" (International System standard) throughout the text and tables.

Response: The authors acknowledge the reviewer comments.

* Units of Measurement: The unit of sintering temperature in Table 2 is changed to "°C" and highlighted in the manuscript.

* Scientific Language: The authors have replaced the informal terms throughout the manuscript and highlighted the same.

* Time Standardisation: The authors thank the reviewer for pointing this out. The abbreviation “hrs” has been replaced with “h” throughout the manuscript in accordance with the International System of Units.

Attachment Submitted filename: Response to reviewers.docx

# Editor report: Decision Letter 2

**DOI:** 10.1371/journal.pone.0357759.r006

**Authors:** Wislei Riuper Osório

9 Aug 2026

Dear Dr. Katiyar,

Thank you for submitting your manuscript to PLOS ONE. After careful consideration, we feel that it has merit but does not fully meet PLOS ONE’s publication criteria as it currently stands. Therefore, we invite you to submit a revised version of the manuscript that addresses the points raised during the review process.

Please submit your revised manuscript by Sep 08 2026 11:59PM. If you will need more time than this to complete your revisions, please reply to this message or contact the journal office at plosone@plos.org. When you're ready to submit your revision, log on to https://www.editorialmanager.com/pone/ and select the 'Submissions Needing Revision' folder to locate your manuscript file.

- A letter that responds to each point raised by the academic editor and reviewer(s). You should upload this letter as a separate file labeled 'Response to Reviewers'.
- A marked-up copy of your manuscript that highlights changes made to the original version. You should upload this as a separate file labeled 'Revised Manuscript with Track Changes'.
- An unmarked version of your revised paper without tracked changes. You should upload this as a separate file labeled 'Manuscript'.

If you would like to make changes to your financial disclosure, please include your updated statement in your cover letter. Guidelines for resubmitting your figure files are available below the reviewer comments at the end of this letter.

If applicable, we recommend that you deposit your laboratory protocols in protocols.io to enhance the reproducibility of your results. Protocols.io assigns your protocol its own identifier (DOI) so that it can be cited independently in the future. For instructions see: https://journals.plos.org/plosone/s/submission-guidelines#loc-laboratory-protocols. Additionally, PLOS ONE offers an option for publishing peer-reviewed Lab Protocol articles, which describe protocols hosted on protocols.io. Read more information on sharing protocols at https://plos.org/protocols?utm_medium=editorial-email&utm_source=authorletters&utm_campaign=protocols.

As the corresponding author, your ORCID iD is verified in the submission system and will appear in the published article. PLOS supports the use of ORCID, and we encourage all coauthors to register for an ORCID iD and use it as well. Please encourage your coauthors to verify their ORCID iD within the submission system before final acceptance, as unverified ORCID iDs will not appear in the published article. *Only*  the individual author can complete the verification step; PLOS staff *cannot*  verify ORCID iDs on behalf of authors.

We look forward to receiving your revised manuscript.

Kind regards,

Wislei Riuper Osório

Academic Editor

PLOS One

Journal Requirements:

If the reviewer comments include a recommendation to cite specific previously published works, please review and evaluate these publications to determine whether they are relevant and should be cited. There is no requirement to cite these works unless the editor has indicated otherwise.

Please review your reference list to ensure that it is complete and correct. If you have cited papers that have been retracted, please include the rationale for doing so in the manuscript text, or remove these references and replace them with relevant current references. Any changes to the reference list should be mentioned in the rebuttal letter that accompanies your revised manuscript. If you need to cite a retracted article, indicate the article’s retracted status in the References list and also include a citation and full reference for the retraction notice.

**Additional Editor Comments:**

Although it is verified that a great number of improvements are incorporated, before its final publication, there exist a MINOR OBLIGATORY revision, as followed by:

1.Table 1 should be reworked and error ranges included. At least the equipment error should be considered.

2. JCPDS file numbers corresponding with XRD patterns should be included.

3. Fig. 2 has no indicating its repeatability or reproducibility. Besides, is a bulk sample utilized or a powder sample is used?

4. Discussion of Figs. 3 and 4, at least a new sentence should be included in order to clarify its reproducibility.

5. The revised text should be verified in order to prevent both US and British English styles. It is verified words in two styles.

[Note: HTML markup is below. Please do not edit.]

[NOTE: If reviewer comments were submitted as an attachment file, they will be attached to this email and accessible via the submission site. Please log into your account, locate the manuscript record, and check for the action link "View Attachments". If this link does not appear, there are no attachment files.]

To ensure your figures meet our technical requirements, please review our figure guidelines: https://journals.plos.org/plosone/s/figures

You may also use PLOS’s free figure tool, NAAS, to help you prepare publication quality figures: https://journals.plos.org/plosone/s/figures#loc-tools-for-figure-preparation.

NAAS will assess whether your figures meet our technical requirements by comparing each figure against our figure specifications.

# Author comment: Author response to Decision Letter 3

**DOI:** 10.1371/journal.pone.0357759.r007

21 Aug 2026

Response to Editor/Reviewers’ comments

Comment 1. Table 1 should be reworked and error ranges included. At least the equipment error should be considered.

Response: We thank the editor for this important suggestion. Table 1 has been revised accordingly, and the corresponding error ranges have been included. The equipment-related measurement errors have been considered and incorporated in the revised table.

Comment 2. JCPDS file numbers corresponding with XRD patterns should be included.

Response: The authors are thankful for the valuable suggestion. The corresponding JCPDS reference card numbers have been added to the revised manuscript for the phases identified from the XRD patterns and relevant reference are also included and highlighted in the manuscript.

Comment 3. Fig. 2 has no indicating its repeatability or reproducibility. Besides, is a bulk sample utilized or a powder sample is used?

Response: We thank the editor for this thoughtful assessment. Five samples were tested for XRD for better repeatability of results. Also, a bulk sintered samples were utilized for the testing. Also, details are now included in manuscript and highlighted the same.

Comment 4. Discussion of Figs. 3 and 4, at least a new sentence should be included in order to clarify its reproducibility.

Response: The authors acknowledge the reviewer comment. The authors have added the sentences in the manuscript and highlighted. To ensure the reproducibility of the microstructural observations, optical and SEM images were obtained from three independently prepared samples under identical experimental and imaging conditions, and the observed microstructural features were found to be consistent across the samples. The details are now included in the manuscript and highlighted the same.

Comment 5. The revised text should be verified in order to prevent both US and British English styles. It is verified words in two styles.

Response: The revised manuscript has been thoroughly checked to ensure consistent use of American English, and all instances of mixed American and British spelling have been corrected accordingly.

Attachment Submitted filename: Response to Editor.docx

# Editor report: Decision Letter 3

**DOI:** 10.1371/journal.pone.0357759.r008

**Authors:** Wislei Riuper Osório

23 Aug 2026

Prediction of the properties of sintered copper-graphite composites using response surface methodology

PONE-D-26-21825R3

Dear Dr. Katiyar,

We’re pleased to inform you that your manuscript has been judged scientifically suitable for publication and will be formally accepted for publication once it meets all outstanding technical requirements.

Within one week, you’ll receive an e-mail detailing the required amendments. When these have been addressed, you’ll receive a formal acceptance letter and your manuscript will be scheduled for publication.

An invoice will be generated when your article is formally accepted. Please note, if your institution has a publishing partnership with PLOS and your article meets the relevant criteria, all or part of your publication costs will be covered. Please make sure your user information is up-to-date by logging into Editorial Manager at Editorial Manager® and clicking the ‘Update My Information' link at the top of the page. For questions related to billing, please contact billing support.

If your institution or institutions have a press office, please notify them about your upcoming paper to help maximize its impact. If they’ll be preparing press materials, please inform our press team as soon as possible -- no later than 48 hours after receiving the formal acceptance. Your manuscript will remain under strict press embargo until 2 pm Eastern Time on the date of publication. For more information, please contact onepress@plos.org.

Kind regards,

Wislei Riuper Osório

Academic Editor

PLOS One

Additional Editor Comments (optional):

Based on the three revised version, it seems that this 3rd version has a great number of improvements in terms of statistical aspects. Associated with this, improvements in organizational stricture and English writing has also been provided. It is also recognized that after three round-revision stages, great efforts were provided by Reviewers to promote suggestions and comments. Rebuttals are provided and majority troubles and reasonably solved and a more soundness and quality revised version is attained. Although, it is still remained “few” weaknesses (grammar and spelling) and scale deficiencies in Figure, which can be solved in “ PROOF VERSION “, the manuscript deserves its final publication. It is important to remark that a MINOR OBLIGATORY revision should be suggested to solve the aforementioned weakness points. However, due to its delaying revision period and based in the fact that Authors surely will be committed with these modifications, the manuscript is approved to its final publication.

Reviewers' comments:

# Editor report: Acceptance letter

**DOI:** 10.1371/journal.pone.0357759.r009

**Authors:** Wislei Riuper Osório

PONE-D-26-21825R3

PLOS One

Dear Dr. Katiyar,

I'm pleased to inform you that your manuscript has been deemed suitable for publication in PLOS One. Congratulations! Your manuscript is now being handed over to our production team.

At this stage, our production department will prepare your paper for publication. This includes ensuring the following:

* All references, tables, and figures are properly cited

* All relevant supporting information is included in the manuscript submission,

* There are no issues that prevent the paper from being properly typeset

You will receive further instructions from the production team, including instructions on how to review your proof when it is ready. Please keep in mind that we are working through a large volume of accepted articles, so please give us a few days to review your paper and let you know the next and final steps.

Lastly, if your institution or institutions have a press office, please let them know about your upcoming paper now to help maximize its impact. If they'll be preparing press materials, please inform our press team within the next 48 hours. Your manuscript will remain under strict press embargo until 2 pm Eastern Time on the date of publication. For more information, please contact onepress@plos.org.

You will receive an invoice from PLOS for your publication fee after your manuscript has reached the completed accept phase. If you receive an email requesting payment before acceptance or for any other service, this may be a phishing scheme. Learn how to identify phishing emails and protect your accounts at https://explore.plos.org/phishing.

If we can help with anything else, please email us at customercare@plos.org.

Thank you for submitting your work to PLOS One and supporting open access.

Kind regards,

PLOS One Editorial Office Staff

on behalf of

Dr. Wislei Riuper Osório

Academic Editor

PLOS One
